# Supplementary material for: Unveiling histotype-specific biomarkers in ovarian carcinoma using proteomics
Source: Mol Ther Oncol. 2025 Jul 16;33(3):201019. doi: 10.1016/j.omton.2025.201019 (PMC12328698; doi:10.1016/j.omton.2025.201019)
Supplement: Document S1. Figure S1, and Tables S1 and S5 [file mmc1.pdf]

OMTON, Volume *33*

Supplemental information

Unveiling histotype-specific biomarkers

in ovarian carcinoma using proteomics

Lucas Werner, Ella Ittner, Hugo Swenson, Elisabeth Werner Rönnerman, Claudia Mateoiu, Anikó Kovács, Pernilla Dahm-Kähler, Per Karlsson, Annika Thorsell, Elham Rekabdar, Parisa Esmaeili, Fredrik Levander, Eva Forssell-Aronsson, Axel Stenmark Tullberg, Ghassan Saed, Toshima Z. Parris, and Khalil Helou

Supplemental Figures

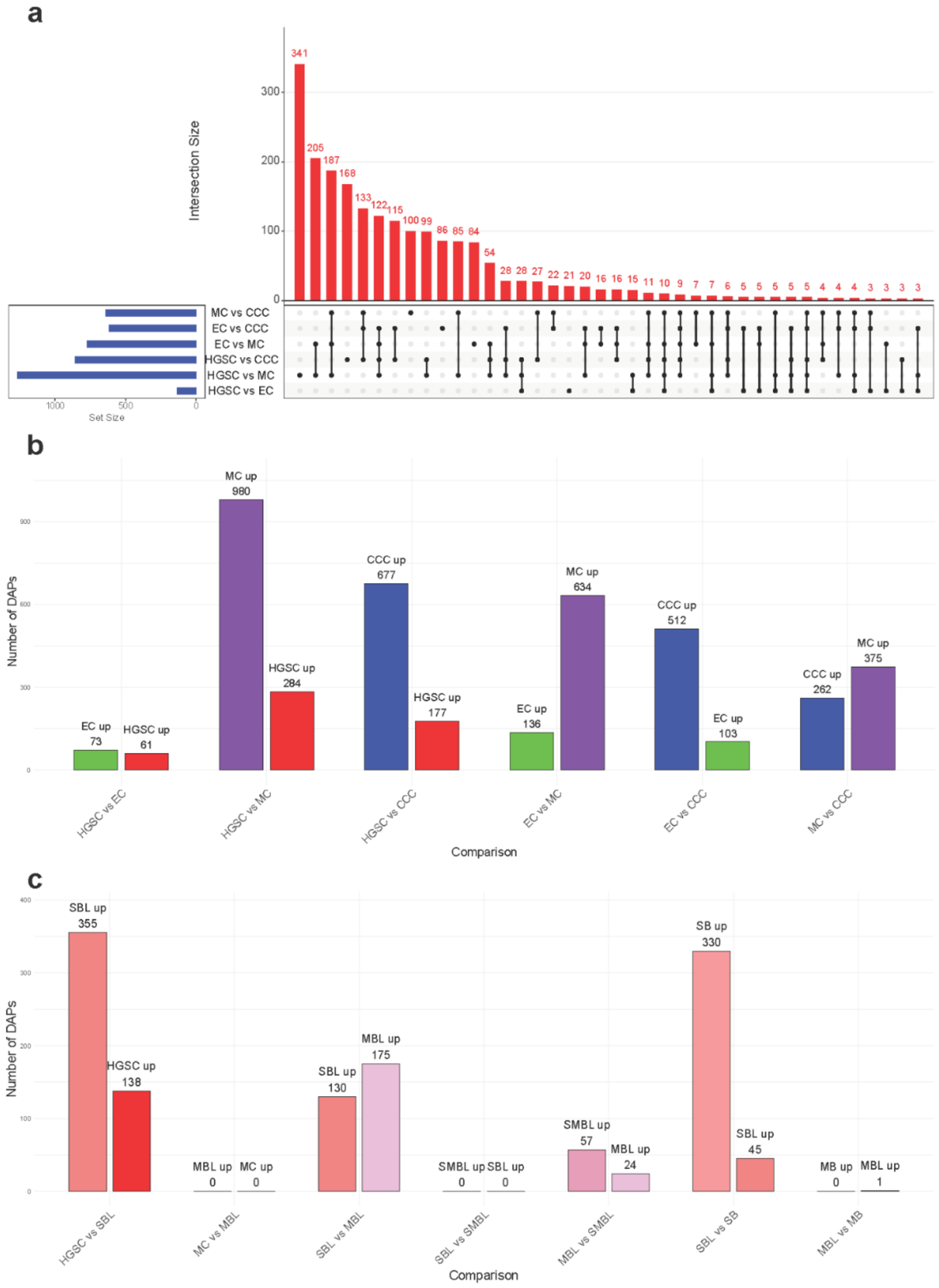

Figure S1

Summary of the differential abundance analysis for pairwise histotype comparisons of malignancies, borderline, benign tumors.

a Upset plot displaying the size distribution of pairwise comparisons along with existing overlaps. **b** Histogram for directionality of abundance for the DAPs for each histotype comparison. **c** Histogram of abundance directionality for HGSC-MC and all BL and BL to B comparisons. B Benign, BL Borderline, CCC Clear-cell ovarian carcinoma, DAP Differentially abundant protein, EC Endometrioid ovarian carcinoma HGSC High-grade serous ovarian carcinoma, MBL Mucinous borderline tumor, MC Mucinous ovarian carcinoma, SBL Serous borderline tumor, SMBL Sero-mucinous borderline tumor.

Supplemental Tables

Table S1

Cohort design and clinicopathologic data after histotype reclassification.

B, Benign; BL, Borderline; CCC, Clear cell ovarian carcinoma; EC, Endometrioid ovarian carcinoma; HGSC, High-grade serous ovarian carcinoma; LGSC, Low-grade serous ovarian carcinoma; MC, Mucinous ovarian carcinoma; NA, Not applicable.

	**HGSC**	**EC**	**MC**	**CCC**	**LGSC**	**BL**	**B**
**Number of samples**	122	42	35	45	8	31	17
**Borderline/benign type**							
Serous						16	11
Mucinous						10	6
Seromucinous						4	0
Endometrioid						1	0
**Patient age**							
Mean	59	61	59	61	54	44	49
Range	34-86	25-86	30-86	40-84	39-77	17-62	23-76
**Overall survival**							
0-2 years	24	9	19	20			
2-5 years	40	13	6	6			
5-10 years	36	8	6	8			
>10 years	22	12	4	11			
**Cause of death**							
Ovarian carcinoma	77	18	19	21			
Other	23	11	13	18			
Alive	22	13	3	6			
**Stage**							
I	26	14	8	12			
II	21	7	2	2			
III	67	16	20	25			
IV	8	5	5	6			
**Dualistic model**							
Type I	0	42	35	45			
Type II	122	0	0	0			
**CA-125**							
<=35	10	2	4	6			
>35	26	4	5	4			
Not answered	48	23	12	9			
Not performed	15	7	6	7			
NA	23	6	8	19			
**Residual tumor size at cytoreductive surgery**							
Tumor free	62	25	14	22			
<=1 cm	21	3	2	2			
>1 cm	29	9	11	8			
Peritoneal carcinosis	8	3	2	1			
NA	2	2	6	12			

Table S2

All unique identified protein accession Ids for searching raw mass spectrometry data with directDIA in Spectronaut along with metadata for all samples.

Table S3
Results from differential abundance analysis after filtering for proteins significantly upregulated (FDR < 0.05, FC > 1.5) and downregulated (FDR < 0.05, FC < -1.5) for all histotypes.

Table S4
Results from differential abundance analysis after filtering for proteins significantly upregulated (FDR < 0.05, FC > 1.5) and downregulated (FDR < 0.05, FC < -1.5) for all pairwise comparisons.

Table S5

Combinations of DAPs yielding the highest AUC for the classification of a histotype/comparison based on SVM models.

Support vecor machine (SVM) models were trained on 80% of the abundance data, and performance tested on the remainingf 20%. SVM models were tested for proteins that had been pre-selected in least absolute shrinkage and selection operator (LASSO) regression, choosing proteins with non-zero coefficients based on lamda min. AUC, Area under the curve; CCC, Clear cell ovarian carcinoma; EC, Endometrioid ovarian carcinoma; HGSC, High-grade serous ovarian carcinoma; MB, Mucinous benign; MBL, Mucinous borderline; MC, Mucinous ovarian carcinoma; SB, Serous benign; SBL, Serous borderline; SMBL, Sero-mucinous borderline.

Panel for histotype/comparison	Protein panel	Protein description panel	AUC
HGSC	GPRC5A, RAB6B, S100A1, CDH6, SSBP1	Retinoic acid-induced protein 3, Ras-related protein Rab-6B, Protein S100-A1, Cadherin-6, Single-stranded DNA-binding protein. mitochondrial	0.854166666666667
EC	ASS1, MAP2K6, PLA2G4A, STRA6	Argininosuccinate synthase, Dual specificity mitogen-activated protein kinase kinase 6, Cytosolic phospholipase A2, Receptor for retinol uptake STRA6	0.804878048780488
MC	KALRN, PPDPF, CALB2, FAM3D, HSPA12A	Kalirin, Pancreatic progenitor cell differentiation and proliferation factor, Calretinin, Protein FAM3D, Heat shock 70 kDa protein 12A	0.840625
CCC	GLRX, PCSK6, ALDH3A2, PLS3, RIMKLB	Glutaredoxin-1, Proprotein convertase subtilisin/kexin type 6, Aldehyde dehydrogenase family 3 member A2, Plastin-3, Beta-citrylglutamate synthase B	0.93287037037037
HGSC vs EC	PDZD8, PTS, VWA2	PDZ domain-containing protein 8, 6-pyruvoyl tetrahydrobiopterin synthase, von Willebrand factor A domain-containing protein 2	0.674285714285714
HGSC vs MC	AGR2, S100A1, PTGS1, KCTD1, BBOX1	Anterior gradient protein 2 homolog, Protein S100-A1, Prostaglandin G/H synthase 1, BTB/POZ domain-containing protein KCTD1, Gamma-butyrobetaine dioxygenase	0.823076923076923
HGSC vs CCC	MET, FAM83B, SPON1, S100A1, CDKN2A	Hepatocyte growth factor receptor, Protein FAM83B, Spondin-1, Protein S100-A1,	0.985714285714286

		Cyclin-dependent kinase inhibitor 2A	
EC vs MC	LYPLAL1, H1-3, CLDN3, CDCA8, KCTD1	Lysophospholipase-like protein 1, Histone H1.3, Claudin-3, Borealin, BTB/POZ domain-containing protein KCTD1	0.767857142857143
EC vs CCC	AP2A2, WARS1, SPATS2, ALDH3A2, CTH	AP-2 complex subunit alpha-2, Tryptophan--tRNA ligase. cytoplasmic, Spermatogenesis-associated serine-rich protein 2, Aldehyde dehydrogenase family 3 member A2, Cystathionine gamma-lyase	0.875
MC vs CCC	AGR3, ST6GALNAC1, CEACAM5, FCGBP, AKR7A3	Anterior gradient protein 3, Alpha-N-acetylgalactosaminide alpha-2.6-sialyltransferase 1, Carcinoembryonic antigen-related cell adhesion molecule 5, IgGFc-binding protein, Aflatoxin B1 aldehyde reductase member 3	0.968253968253968
HGSC vs SBL	SDHAF2, PTBP2, PLBD1, STMN1, PLIN2	Succinate dehydrogenase assembly factor 2. mitochondrial, Polypyrimidine tract-binding protein 2, Phospholipase B-like 1, Stathmin, Perilipin-2	0.945652173913043
SBL vs MBL	S100A1, KLK6, THSD4, CLIC5, KRT17	Protein S100-A1, Kallikrein-6, Thrombospondin type-1 domain-containing protein 4, Chloride intracellular channel protein 5, Keratin. type I cytoskeletal 17	1
MBL vs SMBL	FCGBP, OGN, MAOA, CMBL, PARPBP	IgGFc-binding protein, Mimecan, Amine oxidase [flavin-containing] A, Carboxymethylenebutenolidase homolog, PCNA-interacting partner	1
SBL vs SB	MFSD6, MAPK13, SLC22A18, OSBPL3, TCIRG1	Major facilitator superfamily domain-containing protein 6, Mitogen-activated protein kinase 13, Solute carrier family 22 member 18, Oxysterol-binding protein-related protein 3, V-type proton ATPase 116 kDa subunit a 3	1

Table S6
Significantly enriched (FDR < 0.20) biological processes for the full proteome and for cancer hallmark pathways in gene set enrichment analysis.

Table S7
Significantly enriched (FDR < 0.05) biological processes based on DAPs for respective histotype in a GOEA and number of ocurrences of DAPs.

Table S8

Metadata for survival analysis.

Table S9
All proteins significantly ($p < 0.05$) associated with either high risk ($HR > 1$) or low risk ($HR < 1$) of death form univariate cox regression.

Table S10
Significant proteins from univariate cox regression ($p < 0.05$) that had significant log-rank p-value ($p < 0.05$) in both OS and DSS.

Table S11
Significant ($FDR < 0.05$) proteins for multivariate cox regression for log-rank significant ($p < 0.05$) adjusted for LASSO-selected covariates as well as bootstrap validation of survival models.
