# Supplementary material for: Unveiling histotype-specific biomarkers in ovarian carcinoma using proteomics
Source: Mol Ther Oncol. 2025 Jul 16;33(3):201019. doi: 10.1016/j.omton.2025.201019 (PMC12328698; doi:10.1016/j.omton.2025.201019)
Supplement: Document S2. Article plus supplemental information [file mmc3.pdf]

Molecular Therapy
Oncology
Original Article

Unveiling histotype-specific biomarkers in ovarian carcinoma using proteomics

Lucas Werner,[1,2] Ella Ittner,[1,2] Hugo Swenson,[1,2] Elisabeth Werner Rönnerman,[1,2,3] Claudia Mateoiu,[3] Anikó Kovács,[3,4] Pernilla Dahm-Kähler,[5] Per Karlsson,[1,6] Annika Thorsell,[7] Elham Rekabdar,[7] Parisa Esmaeili,[8] Fredrik Levander,[8] Eva Forssell-Aronsson,[2,9,10] Axel Stenmark Tullberg,[1,11] Ghassan Saed,[12,13,14] Toshima Z. Parris,[1,2] and Khalil Helou[1,2]

[1]Department of Oncology, Institute of Clinical Sciences, Sahlgrenska Academy, University of Gothenburg, 41390 Gothenburg, Sweden; [2]Sahlgrenska Center for Cancer Research, Sahlgrenska Academy, University of Gothenburg, 41390 Gothenburg, Sweden; [3]Region Västra Götaland, Sahlgrenska University Hospital, Department of Clinical Pathology, 41345 Gothenburg, Sweden; [4]Institute of Biomedicine, Sahlgrenska Academy, University of Gothenburg, 40530 Gothenburg, Sweden; [5]Department of Obstetrics and Gynecology, Institute of Clinical Sciences, Sahlgrenska Academy at University of Gothenburg, 41685 Gothenburg, Sweden; [6]Region Västra Götaland, Sahlgrenska University Hospital, Department of Oncology, 41345 Gothenburg, Sweden; [7]Proteomics Core Facility at Sahlgrenska Academy, University of Gothenburg, 40530 Gothenburg, Sweden; [8]Department of Immunotechnology, Lund University, 22100 Lund, Sweden; [9]Department of Medical Radiation Sciences, Institute of Clinical Sciences, Sahlgrenska Academy, University of Gothenburg, 41345 Gothenburg, Sweden; [10]Region Västra Götaland, Sahlgrenska University Hospital, Department of Medical Physics and Biomedical Engineering, 41345 Gothenburg, Sweden; [11]Department of Medicine, Stanford University, Stanford, CA 94305, USA; [12]Department of Obstetrics and Gynecology, Wayne State University School of Medicine, Detroit, MI 48201, USA; [13]Department of Gynecologic Oncology, Karmanos Cancer Institute, Detroit, MI 48201, USA; [14]Department of Obstetrics and Gynecology, University of Jordan School of Medicine, Amman 11942, Jordan

Epithelial ovarian cancer (EOC) is the most lethal gynecologic malignancy, yet clinical tools for diagnosis, prognosis, and treatment remain limited, and molecular profiling of histotypes is lacking. Here, we leverage proteomic data to further stratify four main EOC histotypes, borderline (BL) and benign (B) tumors, and identify candidate prognostic and diagnostic biomarkers. Using proteomic data from 300 patient samples, we identified differentially abundant proteins (DAPs) such as SNCG, S100A1, VWA2, AGR2, CTH, and SPINK1 and biomarker panels to stratify the tissues. Enrichment of biological processes profiled histotypes and involvement of DAPs. Survival analysis identified candidate biomarkers predicting overall- and disease-specific survival with histotype-specificity. Of these, GLYR1, RPL12, GDPGP1, and POLR2M were associated with favorable outcomes, while SDF4, PPP3CC, EIF2AK2, and STX6 were linked to unfavorable outcomes. Collectively, these findings provide histotype-specific attributes for known and EOC biomarkers that may serve as clinical tools for EOC diagnosis and treatment decisions.

INTRODUCTION

Ovarian cancer is an umbrella term for a multitude of malignancies that originate not only from the ovaries, but also from other surrounding tissues such as the fallopian tubes. Histologically, the disease arises from the epithelium, stroma, or germ cells, of which epithelial malignancies being by far the most prevalent.[1] It is a disease characterized by diagnosis at a late stage and poor survival rate, being the most lethal gynecologic malignancy.[2,3] Over the years, the understanding of the molecular and histopathological characteristics of epithelial ovarian cancer (EOC) has improved. Epithelial ovarian tumors are classified based on their morphological features into benign (B), borderline (BL), and malignant subtypes. This classification reflects differences in biological behavior, histopathological characteristics, and clinical outcomes. This has led to the establishment of five main histotypes: high-grade serous (HGSC), low-grade serous (LGSC), endometrioid (EC), mucinous (MC), and clear-cell (CCC) ovarian carcinoma, as stated in the current 2020 World Health Organization (WHO) criteria.[4] Despite differences in biological and clinical features between the histotypes, diagnosis and standard treatment for EOC patients remains largely the same regardless of histotype.[4–6]

Diagnostic methods are limited. CA-125 is the only FDA-approved serum biomarker to diagnose and monitor EOC, with HE4 used as a complementary biomarker.[6] However, only about half of patients diagnosed at stage I display elevated CA-125 levels. Moreover, CA-125 is also overexpressed in other cancers, such as hematological malignancies and colorectal cancer, but also in some patients without cancer.[7–9] Furthermore, as early-stage EOC is often asymptomatic, symptoms are typically vague; over 70% of EOCs are diagnosed at a late stage.[10] Therefore, there is an urgent need for prognostic, diagnostic, and therapeutic biomarkers to guide personalized treatment

Received 13 May 2025; accepted 10 July 2025;
https://doi.org/10.1016/j.omton.2025.201019.

Correspondence: Lucas Werner, Department of Oncology, Institute of Clinical Sciences, Sahlgrenska Academy, University of Gothenburg, Medicinaregatan 1F, SE-413 90 Gothenburg, Sweden.
E-mail: lucas.werner@gu.se

© 2025 The Author(s). Published by Elsevier Inc. on behalf of The American Society of Gene and Cell Therapy. This is an open access article under the CC BY license (http://creativecommons.org/licenses/by/4.0/).

plans and improve disease stratification based on clinicopathological features.

Current prognostic tools are lacking histotype-specific characterization.[11] Furthermore, specific treatment regimens based on histotype have not been implemented. For more than two decades, cytoreductive surgery followed by platinum-based chemotherapy has remained the standard treatment. Some improvements have been made. Patients carrying genetic aberrations in BRCA1/2 receive targeted therapy with poly ADP-ribose polymerase inhibitors as maintenance therapy, which has been shown to improve progression-free survival, most notably for HGSC patients.[12,13] Although more personalized treatment approaches such as stage-based treatment plans and the use of angiogenesis inhibitors are emerging, most display no significant improvement in survival. Following conventional therapy, 5- and 10-year survival rates in Sweden are currently at 60% and 48%, respectively (2023).[14] In all, there is therefore an urgent need for additional prognostic, diagnostic, and therapeutic biomarkers to guide personalized treatment plans and improve disease stratification based on clinicopathological features.

Currently, established biomarkers for molecular subtyping of the five main histotypes serve as a complement to traditional morphological histotype-profiling.[4] However, research efforts devoted to identifying biomarkers have largely focused on distinguishing between B, BL, and malignancies.[15,16] In recent years, substantial efforts have been made to explore the proteome of EOC. Qian et al. derived diagnostic and prognostic candidates while also mapping distinct biological processes associated with respective histotype by studying the proteome of ovarian tissues.[17] Other studies have contributed similarly with tools for stratifying EOC.[18] However, there is still a lack of emphasis on establishing distinct biomarkers specific for each histotype and tissue type, as research has either focused on one histotype or grouped them as one entity when studying differences in protein abundance between tissues.

Therefore, in this study, we used liquid chromatography-mass spectrometry on primary EOCs, BL, and B ovarian tumors to study the proteomic landscape. By analyzing the abundance profiles of the tissues, we aim to uncover highly up- and downregulated proteins and derive combinations of these that can be used to stratify histotypes, BL, and B that can provide complementary panels to established histopathology and used to further investigate potential diagnostic tools. To uncover the biological signatures for the histotypes and identify the involvement of dysregulated proteins, we perform enrichment analyses of biological processes to uncover pathways that may be involved in the development and progression of each histotype. Lastly, survival analysis aims to identify proteins associated with high and low risk of death, providing prognostic tools for EOC histotypes.

RESULTS

Proteomic analysis and protein quantification

To explore the proteomic profiles in the EOC patients and to identify biomarkers, four-dimensional data independent acquisition (4D-DIA) proteomics was employed for in-depth profiling. The cohort comprised 300 patients. Of the main histotypes, patients were diagnosed with HGSC (n = 122), LGSC (n = 8), EC (n = 42), MC (n = 35), and CCC (n = 45). BL material consisted of serous (n = 16), MC (n = 10), seromucinous (n = 4), and EC (n = 1) tissues. B tissue covered cases of serous (n = 11) and MC (n = 6) type. LGSC and EC BL were excluded due to low sample counts. A detailed overview of the cohort and the clinical characteristics are shown in Table S1. Analysis of the tissue samples retrieved 12,707 protein groups, with 10,308 unique protein groups considered for quantitative evaluation after filtering for the detected abundance in at least 70% of samples in at least one histotype (Table S2).

Differential abundance analysis

To identify proteins that may be capable of stratifying EOC histotypes, analysis of false discovery rate (FDR) and fold changes (FCs) between abundances was employed to find proteins of significant up- or downregulation (FDR < 0.05, |FC| > 1.5) in a histotype compared to the others, differentially abundant proteins (DAPs). This generated 494, 9, 551, and 487 DAPs for HGSC, EC, MC, and CCC, respectively (Figure 1A; Table S3). The DAPs were largely histotype-specific, with some overlaps between them (Figure 1B). FCs suggested that the strongest upregulated protein specific for HGSC was S100A1, appearing to also be the most downregulated in MC. AGR2 may be a strong candidate for MC-specificity with high upregulation, while being the most downregulated protein in HGSC. Similarly, SPINK1 was one of the most upregulated proteins in MC, while being the most downregulated in HGSC and CCC. VWA2 and CTH showed uniquely high upregulation in EC and CCC, respectively (Figures 1C–1F). FDR and log2 FC for the most up- and downregulated proteins for each histotype, along with their protein symbol and description, can be found in Table 1.

Pairwise differential abundance analysis

Pairwise analysis of differential abundance (DA) across all histotype comparisons revealed protein signatures for each. The highest number of DAPs was found between HGSC and MC (n = 1,264), and the fewest between HGSC and EC (n = 134), indicating greater similarity between HGSC and EC (Table S4). Notably, SNCG was specifically upregulated in HGSC compared to EC and CCC, while SPINK1 was strongly elevated in EC versus HGSC and also upregulated in MC compared to CCC. TFF1 and PRODH were the top DAPs in EC vs. MC and EC vs. CCC, respectively. Pairwise analyses also identified unique DAPs for comparisons involving BL and B tissues (Table 2); MX1 and SPATA18 distinguished HGSC from serous borderline (SBL), while KRT23 and OGN differentiated SBL from serous benign (SB). S100A1 was consistently upregulated in serous vs. MC tissue, including both malignant and BL comparisons (Figures S1A and S1B).

While DAPs such as MX1 and STATA18 were identified for HGSC vs. SBL, none for MC vs. mucinous borderline (MBL) were found. In contrast, MBL had DAPs when compared to SBL (S100A1, FCGBP), sero-mucinous borderline (SMBL; AKR1B10, CAPS), and mucinous benign tissue (MB; BAIA2PL1), while it was not possible to

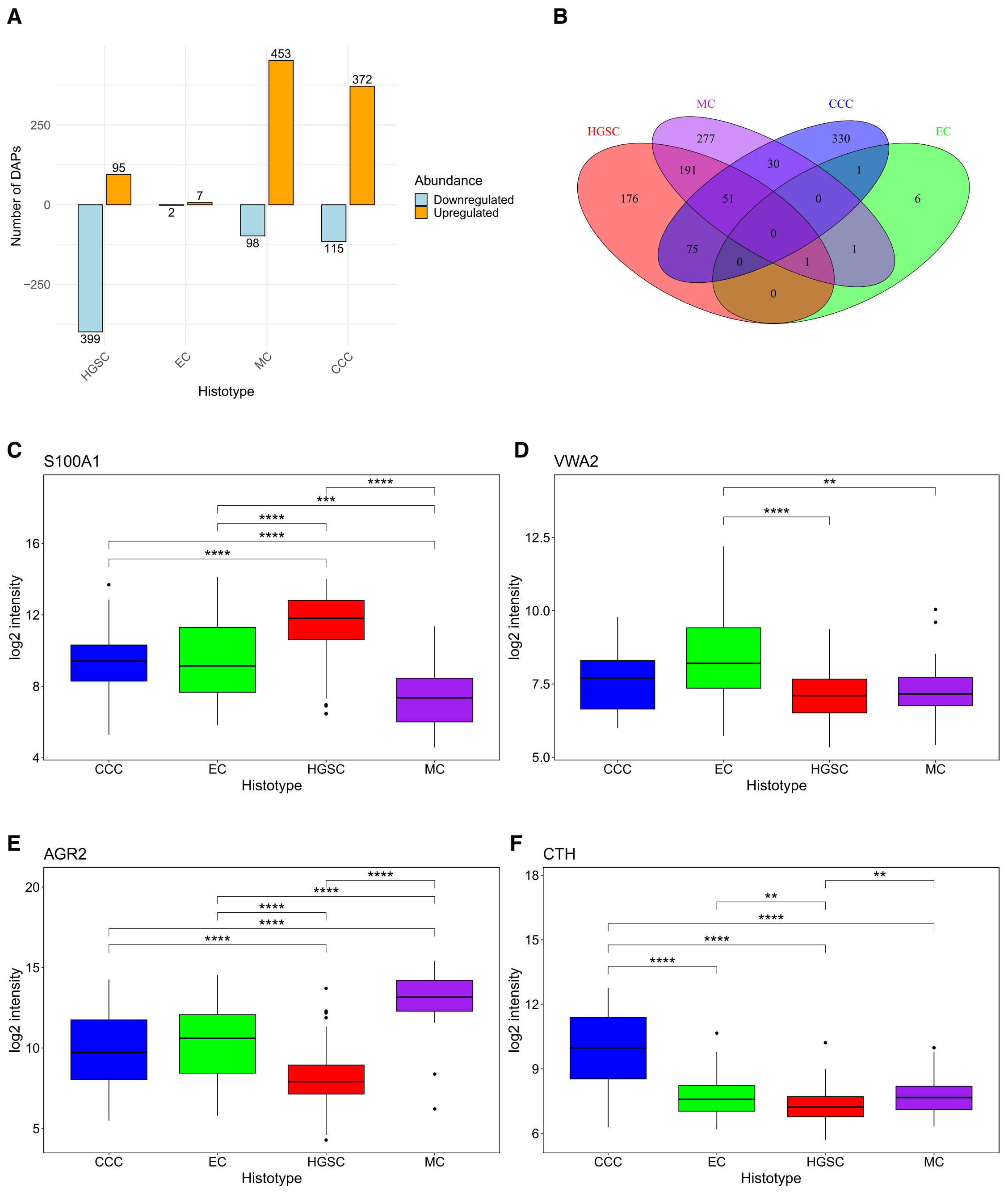

Figure 1. Differential abundance analysis of epithelial ovarian cancer reveals histotype-specific up- and downregulated proteins
(A) Quantification and directionality of the DAPs. (B) A Venn diagram illustrating the overlap of the identified DAPs between the histotypes. (C–F) Boxplots for the most upregulated protein based on log2 FC for (C) HGSC, (D) EC, (E) MC, and (F) CCC. The *p* values were derived from pairwise Wilcoxon tests and FDR corrected. The middle horizontal lines represent the median of the log2 intensity, upper and lower bounds the 25th and 75th percentiles, whiskers the largest intensities 1.5 times outside the percentiles and dots outliers outside these ranges. CCC, clear-cell ovarian carcinoma; DAPs, differentially abundant proteins; EC, endometrioid ovarian carcinoma; FC, fold change; FDR, false discovery rate; HGSC, high-grade serous ovarian carcinoma; MC, mucinous ovarian carcinoma. **FDR $\leq$ 0.01, ***FDR $\leq$ 0.001, and ****FDR $\leq$ 0.0001.

distinguish SBL from SMBL (Figure S1C; Table S4). Like the abundances observed between malignancies, HGSC displayed more downregulation when compared to SBL, whereas MBL had slightly more upregulated DAPs compared to SBL, mirroring the trend observed in HGSC vs. MC. The most DAPs for these comparisons are summarized in Table 2.

Table 1. Most differentially abundant proteins that are histotype-specific

Histotype	Protein accession	Protein symbol	Protein description	FDR	log2 FC
Upregulated					
HGSC	P23297	S100A1	protein S100-A1	6.46e−14	2.14
	P29373	CRABP2	cellular retinoic acid-binding protein 2	7.09e−21	1.94
	O76070	SNCG	gamma-synuclein	1.18e−15	1.87
	P42771	CDKN2A	cyclin-dependent kinase inhibitor 2A	3.27e−15	1.49
	Q96RQ9	IL4I1	L-amino-acid oxidase	6.02e−10	1.36
EC	Q5GFL6	VWA2	von Willebrand factor A domain-containing protein 2	4.23e−5	1.16
	Q9ULR3	PPM1H	protein phosphatase 1H	4.23e−5	0.84
	Q9BX79	STRA6	receptor for retinol uptake STRA6	0.04	0.84
	A1L020	MEX3A	RNA-binding protein MEX3A	0.01	0.77
	Q8IV56	PRR15	proline-rich protein 15	0.02	0.75
MC	O95994	AGR2	anterior gradient protein 2 homolog	5.17e−15	3.73
	P00995	SPINK1	serine protease inhibitor Kazal-type 1	5.16e−5	2.78
	P56470	LGALS4	galectin-4	1.05e−9	2.76
	Q6UX06	OLFM4	olfactomedin-4	9.43e−9	2.61
	P01833	PIGR	polymeric immunoglobulin receptor	1.42e−8	2.58
CCC	P32929	CTH	cystathionine gamma-lyase	1.60e−33	2.41
	O75610	LEFTY1	left-right determination factor 1	1.77e−6	2.30
	Q93099	HGD	homogentisate 1.2-dioxygenase	1.06e−9	1.98
	O43272	PRODH	proline dehydrogenase 1. mitochondrial	2.65e−15	1.97
	Q8IZV5	RDH10	retinol dehydrogenase 10	2.67e−20	1.97
Downregulated					
HGSC	P00995	SPINK1	serine protease inhibitor Kazal-type 1	4.74e−5	−3.00
	O95994	AGR2	anterior gradient protein 2 homolog	1.17e−19	−2.71
	P14091	CTSE	cathepsin E	1.28–6	−2.19
	Q9BYZ8	REG4	regenerating islet-derived protein 4	0.03	−2.12
	P01833	PIGR	polymeric immunoglobulin receptor	1.13e−14	−2.12
EC	E9PAV3	NACA	nascent polypeptide-associated complex subunit alpha. muscle-specific form	0.05	−0.77
	P00966	ASS1	argininosuccinate synthase	0.04	−0.75
MC	P23297	S100A1	protein S100-A1	9.51e−10	−2.97
	P50895	BCAM	basal cell adhesion molecule	6.67e−12	−1.87
	P42771	CDKN2A	cyclin-dependent kinase inhibitor 2A	2.07e−6	−1.62
	Q7Z7D3	VTCN1	V-set domain-containing T cell activation inhibitor 1	0.01	−1.53
	O95436	SLC34A2	sodium-dependent phosphate transport protein 2B	2.64e−4	−1.51
CCC	P00995	SPINK1	serine protease inhibitor Kazal-type 1	0.01	−1.95
	O76070	SNCG	gamma-synuclein	1.31e−6	−1.67
	P06731	CEACAM5	carcinoembryonic antigen-related cell adhesion molecule 5	0.05	−1.56
	P29373	CRABP2	cellular retinoic acid-binding protein 2	8.98e−7	−1.53
	Q9HCB6	SPON1	spondin-1	8.46e−6	−1.50

Five proteins (except downregulated proteins for EC) of highest log2 fold changes for up- and downregulation for each histotype. CCC, clear-cell ovarian carcinoma; EC, endometrioid ovarian carcinoma; FDR, false discovery rate; HGSC, high-grade serous ovarian carcinoma; MC, mucinous ovarian carcinoma; log 2 FC, log2 fold change. Displayed log2 FC values for the protein for a histotype when compared to the abundance of the other histotypes. FDR is based on *p* values from t tests using limma.

Table 2. Most overall and comparison-specific differentially abundant proteins for all pairwise comparisons

Comparison	Upregulated in	Protein accession	Protein symbol	Protein description	FDR	| Log2 FC |
Overall						
HGSC vs. EC	HGSC	O76070	SNCG	gamma-synuclein	4.48e−8	2.06
	EC	P00995	SPINK1	serine protease inhibitor Kazal-type 1	0.02	3.17
HGSC vs. MC	HGSC	P23297	S100A1	protein S100-A1	2.20e−17	3.90
	MC	O95994	AGR2	anterior gradient protein 2 homolog	1.65e−27	4.91
HGSC vs. CCC	HGSC	O76070	SNCG	gamma-synuclein	1.11e−14	2.53
	CCC		LEFTY1	left-right determination factor 1	3.96e−6	2.94
EC vs. MC	EC	P23297	S100A1	protein S100-A1	8.18e−4	1.93
	MC	P04155	TFF1	trefoil factor 1	0.01	2.99
EC vs. CCC	EC	Q14508	WFDC2	WAP four-disulfide core domain protein 2	4.00e−3	1.62
	CCC	O43272	PRODH	proline dehydrogenase 1. Mitochondrial	4.25e−10	2.17
MC vs. CCC	MC	P00995	SPINK1	serine protease inhibitor Kazal-type 1	3.73e−6	3.50
	CCC	Q9Y617	PSAT1	phosphoserine aminotransferase	4.08e−17	2.78
Comparison-specific						
HGSC vs. EC	HGSC	Q8WU39	MZB1	marginal zone B- and B1-cell-specific protein	0.01	1.36
	EC	Q9C040	TRIM2	tripartite motif-containing protein 2	6.47e−9	0.84
HGSC vs. MC	HGSC	P29762	CRABP1	cellular retinoic acid-binding protein 1	0.03	1.58
	MC	Q16853	AOC3	membrane primary amine oxidase	3.00e−3	1.35
HGSC vs. CCC	HGSC	P50238	CRIP1	cysteine-rich protein 1	5.12e−6	1.15
	CCC	P09466	PAEP	glycodelin	0.02	1.10
EC vs. MC	EC	CYP4X1	CYP4X1	cytochrome P450 4X1	0.04	0.88
	MC	Q53GG5	PDLIM3	PDZ and LIM domain protein 3	0.04	0.92
EC vs. CCC	EC	Q9P1F3	ABRACL	costars family protein ABRACL	5.14e−8	0.86
	CCC	P13640	MT1G	metallothionein-1G	0.03	1.49
MC vs. CCC	MC	P02452	COL1A1	collagen alpha-1(I) chain	0.01	0.93
	CCC	O15121	DEGS1	sphingolipid delta(4)-desaturase DES1	3.02e−11	0.96
HGSC vs. SBL	HGSC	P20591	MX1	interferon-induced GTP-binding protein Mx1	5.69e−6	2.57
	SBL	Q8TC71	SPATA18	mitochondria-eating protein	5.69e−6	2.84
SBL vs. MBL	SBL	P23297	S100A1	protein S100-A1	2.00e−3	4.21
	MBL	Q9Y6R7	FCGBP	IgGFc-binding protein	2.196e−5	4.07
MBL vs. SMBL	MBL	O60218	AKR1B10	aldo-keto reductase family 1 member B10	0.01	4.19
	SMBL	Q13938	CAPS	calcyphosin	0.01	4.82
MBL vs. MB	MBL	Q9UHR4	BAIAP2L1	brain-specific angiogenesis inhibitor 1-associated protein 2-like protein 1	0.02	2.20
	MB	–	–	–	–	–
SBL vs. SB	SBL	Q9C075	KRT23	keratin. type I cytoskeletal 23	0.02	2.11
	SB	P20774	OGN	mimecan	0.02	2.66

Proteins of highest log2 fold changes for up- and downregulation for all pairwise comparisons, including a subset of proteins unique for each comparison. CCC, clear-cell ovarian carcinoma; EC, endometrioid ovarian carcinoma; FDR, false discovery rate adjusted for multiple testing with Benjamini-Hochberg model; HGSC, high-grade serous ovarian carcinoma; log2 FC, log2 fold change; MB, mucinous benign; MBL, mucinous borderline tumor; MC, mucinous ovarian carcinoma; SBL, serous borderline tumor; SMBL, seromucinous borderline tumor. FDR is based on p values from t tests using limma.

Protein panels to distinguish between tissues

To find the best combination of DAPs that can be used to distinguish a histotype from others, stepwise support vector machine (SVM) models were used to iteratively add proteins to classification models to find the best sets of proteins that can separate a histotype based on protein abundance. The SVM models were trained and tested on unseen data for proteins that were pre-selected using least absolute shrinkage and selection operator (LASSO) with 5-fold

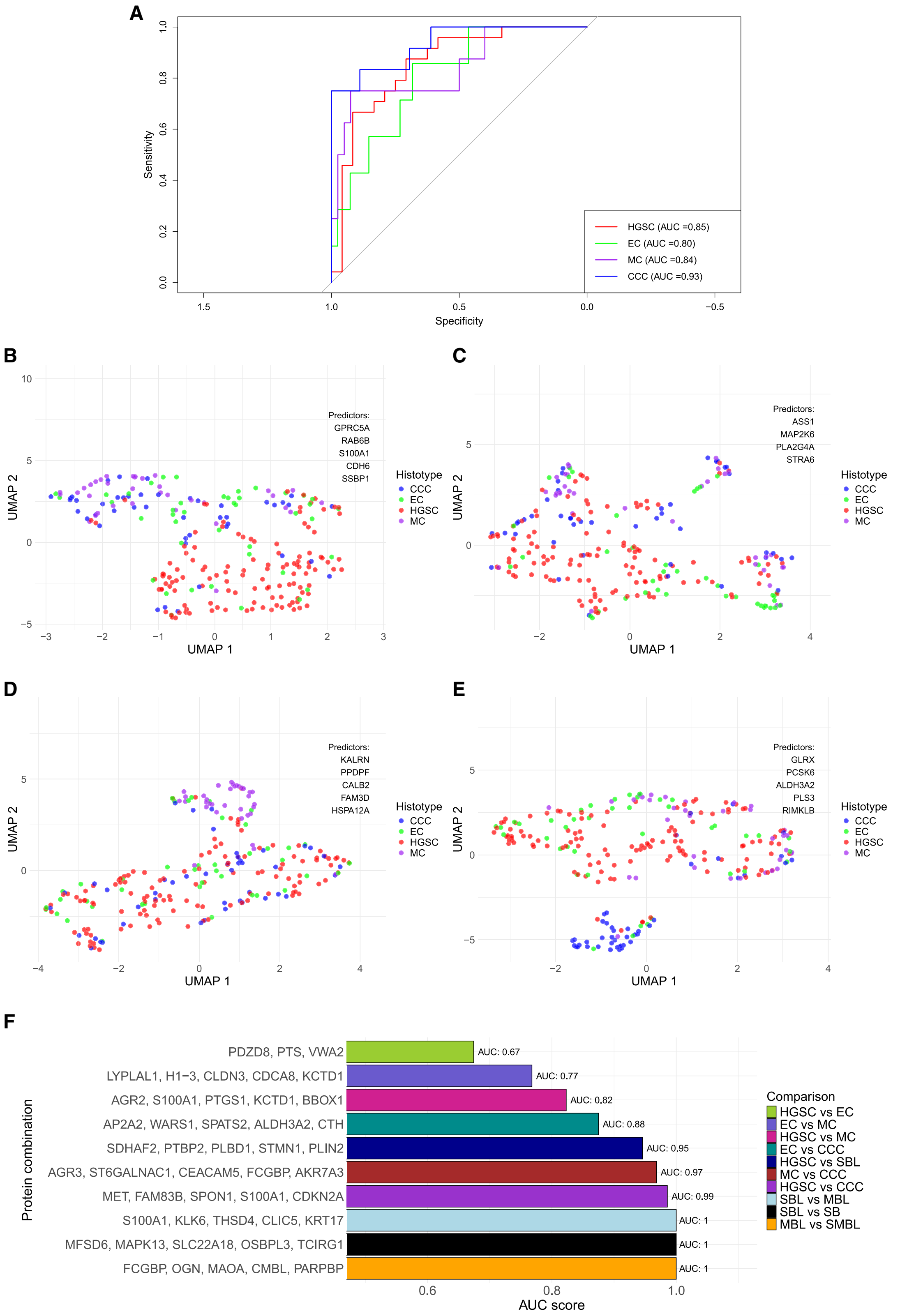

(legend on next page)

cross-validation. The analysis identified panels for all four histotypes. For HGSC, the combination of GPRC5A, RAB6B, S100A1, CDH6, and SSBP1 achieved an area under the curve (AUC) score of 0.85. ASS1, MAP2K6, PLA2G4A, and STRA6 with AUC score 0.80 were deemed the best combination for EC. A classification model with KALRN, PPDPF, CALB2, FAM3D, and HSPA12A was optimal for MC (AUC = 0.84) and GLRX, PCSK6, ALDH3A2, PLS3, and RIMKLB for CCC (AUC = 0.93; Figure 2A; Table S5). Uniform manifold approximation and projections (UMAPs) for the identified protein combinations for respective histotype indicated tight abundance clusters for MC and CCC, whereas abundance of proteins saw more heterogeneous abundance between samples for HGSC and EC, displaying more overlapping with abundances with the other histotypes as well (Figures 2B–2E). Notably, the protein panel for EC showed poor separation of EC samples from samples of other histotypes (Figure 2C).

Protein combinations for all pairwise comparisons were identified as well, except for MBL vs. MB due to few DAPs being identified. These panels are presented in Figure 2F. Numerous highly up- and downregulated DAPs were found in these prediction models. Notably, S100A1 was included for all comparisons with HGSC except for HGSC vs. EC, as well as for SBL vs. MBL. AGR2 was included in the protein set to distinguish MC from HGSC. The most upregulated protein in EC, VWA2, was found to be important for separating EC and HGSC. In all, the models that aimed to stratify histotypes saw the highest predictive performance (AUC score) for HGSC vs. CCC, and the poorest performance for HGSC vs. EC. Near perfect classification was found for SBL vs. MBL, MBL vs. SMBL, and SBL vs. SB, with models consisting of proteins, apart from S100A1, unique compared to all models for the malignancies.

Gene set enrichment analysis

Gene set enrichment analysis (GSEA) on ranked proteins of the full proteome (non-significant and DAPs) for each histotype, identified enriched mutual and unique biological processes.[19] Based on the top five normalized enrichment scores (NES) for both up- and downregulation of significantly enriched biological processes (FDR < 0.05), MC and CCC shared downregulation in RNA splicing events via transesterification, which in contrast was found to be upregulated in EC and not significantly enriched in HGSC. Complement activation was found to be upregulated in CCC, whereas in HGSC and EC this process was mutually downregulated. Processes related to DNA-templated replication and transcription were upregulated in HGSC and EC, but not in MC nor CCC (Figure 3A).

Filtering for uniquely significantly enriched biological processes for the histotypes, up- and downregulated traits could be found (Table S6). Generally, HGSC presented upregulation of antigen processing and presentation, and downregulation of multiple distinct biological processes. This effect was strongest for cilium assembly. Uniquely for EC was upregulation of lung cell differentiation and downregulation of stress response to metal ion. MC was characterized by upregulation of response to gram-negative bacterium and innate immune response in mucosa, whereas multiple transport of nuclear elements processes was downregulated. In CCC, nucleoside catabolic process was distinctly upregulated and regulation of ncRNA transcription was downregulated (Figure 3B).

GSEA for cancer hallmark pathways from MSigDB revealed biological processes that were shared among the histotypes but had distinct regulation patterns.[20] The key identified pathways were E2F targets, MYC targets V1 and V2, G2M checkpoint, xenobiotic metabolism, and epithelial mesenchymal transition. HGSC and EC showed upregulation of E2F targets and MYC targets V1 and V2. These processes were downregulated in MC and CCC. Xenobiotic metabolism was upregulated and G2M checkpoint downregulated in MC and CCC, whereas xenobiotic metabolism saw downregulation in HGSC and G2M checkpoint was upregulated in EC. MC and CCC also shared upregulation of complement. HGSC and EC were also characterized by strong downregulation of epithelial mesenchymal transition, a process that was among the most upregulated in MC (Figure 3C).

Gene ontology enrichment analysis

Gene ontology enrichment analysis (GOEA) of the DAPs revealed the most significantly enriched (FDR < 0.05) biological processes (BPs) driven by the DAPs for respective histotype. Most significant process for HGSC was negative regulation of peptidase activity with 24 DAPs involved, a process also highly enriched in MC. Of the most significantly enriched biological processes in EC, all were related to prostaglandin and eicosanoid secretion with PLA2G4A and MAP2K6 driving the enrichment. MC had highest enrichment of digestion and digestive systems, with very similar proteins involved in the two processes. Extracellular and external encapsulating structure organization as well as extracellular matrix organization were the most significantly enriched processes in CCC, with over 30 DAPs driving the processes (Figure 4A). The complete panels of DAPs involved in the enriched BPs can be found in Table S7. In general, proteins from the serpin-family were commonly identified in the most significantly enriched processes for HGSC. In EC, the equivalent was PLA2G4A and MAP2K6. For MC, proteins

Figure 2. Panels of proteins stratifying histotypes, borderline, and benign tumors with highest predictive power

(A) ROC curve for the combination of DAPs yielding highest AUC score for the four main histotypes. (B–E) UMAPs showing clustering of the patient samples for the listed DAPs (predictors) of highest AUC score for (B) HGSC, (C) EC, (D) MC, and (E) CCC. (F) Barplot displaying the equivalent DAP panels for all pairwise comparisons where sufficient abundance data to build SVM models could be obtained. Differential abundance analysis of MC vs. MBL, SMBL vs. MB, SMBL vs. SB, and MBL and MB generated insufficient amounts of DAPs for SVM modeling and were therefore excluded. AUC, area under the curve; CCC, clear-cell ovarian carcinoma; DAPs, differentially abundant proteins; EC, endometrioid ovarian carcinoma; HGSC, high-grade serous ovarian carcinoma; MBL, mucinous borderline tumor; MC, mucinous ovarian carcinoma; ROC, receiver operating characteristics; SB, serous benign tumor; SBL, serous borderline tumor; SMBL, sero-mucinous borderline; UMAP, uniform manifold approximation and projection.

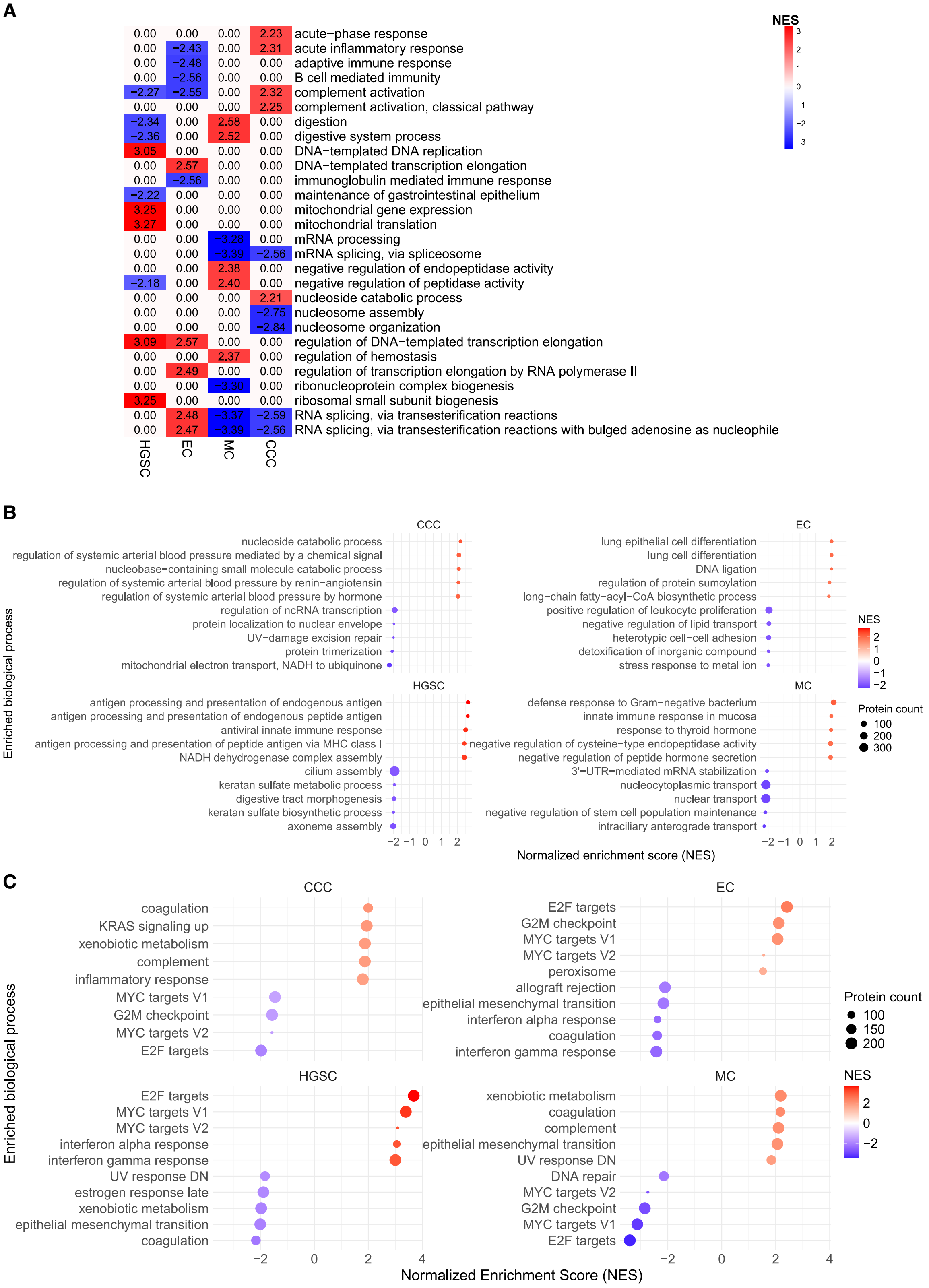

(legend on next page)

from the mucin (MUC)-family were common. The proteins involved in processes for CCC were more heterogeneous, but collagen (COL)-proteins were frequently found.

The DAPs driving the most significantly enriched BPs tended to not correlate with the most up- and downregulated proteins identified in the DA analysis, not even for EC that had comparably few DAPs in respect to the other histotypes. However, SPINK1 that was highly up-regulated in MC and the most downregulated in HGSC and CCC was in this enrichment analysis found to be involved in digestion and digestive systems process for MC. Counting the occurrences of the DAPs for all significantly enriched biological processes for respective histotype revealed PLA2G4A, THBS1, ADA, and SERPING1 to be the most involved proteins for EC, MC, CCC, and HGSC, respectively. Of the most involved proteins, the most upregulated protein in EC, VWA2 was one of them (Figure 4B; Table S7).

Survival analysis

Survival models were constructed to identify proteins showing strongest association with increased and decreased risk of death by utilizing univariate cox regression revealed proteins significantly associated ($p < 0.05$) with overall survival (OS) and disease-specific survival (DSS). The univariate cox proportional hazard (PH) models generated significant proteins for HGSC (385 for OS, 448 for DSS), EC (602 for OS, 780 for DSS), MC (962 for OS, 1,476 for DSS), and CCC (644 for OS, 474 for DSS; Table S8). Log rank tests filtering ($p < 0.05$) followed by multivariate cox regression using LASSO to select and adjust for covariates (stage, age, CA125 levels, and residual tumor size at cytoreductive surgery) yielded proteins with hazard ratios (HRs) indicating increased risk (HR > 1) and decreased risk (HR < 1) of death for patients for both OS and DSS after validating the robustness of the models with bootstrapping (FDR < 0.05, bootstrap p value < 0.2; Tables S9–S11).

Filtering for proteins with the lowest and highest HR while retaining the smallest confidence intervals revealed proteins with the largest estimated impact on observed decreased and increased risk of death when adjusting for the covariates. For the proposed panel for OS, proteins with strongest association with favorable prognosis were UBL4A in HGSC, NDUFS1 in EC, PDE12 in MC, and POLR2M in CCC. The equivalent top candidates for DSS were GLYR1 for HGSC, RPL11 for EC, GDPGP1 for MC, and TEPSIN for CCC (Figures 5A and 5B). For poor prognosis, the candidates for OS were SDF4, PPP3CC, EIF2AK2, and STX6 for HGSC, EC, MC, and CCC, respectively, based on highest adjusted HR. Moreover, poor prognosis for DSS suggested SDF4 for HGSC as in OS, CNN1 for EC, ANK2 for MC, and ATRAID for CCC (Figures 6A and 6B).

DISCUSSION

To explore the proteomic landscape of EOC and identify biomarkers with the potential to stratify EOC histotypes, BL and B tumors as well as yield prognostic and diagnostic biomarker candidates, we employed liquid chromatography-mass spectrometry (LC-MS) to establish a proteome. We performed DA analysis to identify highly up- and downregulated proteins for each histotype and all pairwise comparisons. Our study showed that there are deregulated proteins between the histotypes. Pairwise comparisons showed high and low abundance-proteins for comparisons with BL and B tissues as well while also highlighting that HGSC appear to have comparable protein abundance to EC. GSEA and GOEA found shared and uniquely enriched biological processes for each histotype, with GOEA mapping the involvement of DAPs. Finally, the study of proteins associated with survival revealed biomarkers associated with favorable and unfavorable outcome for both OS and DSS for respective histotype.

The identified deregulated proteins for the histotypes have shown potential clinical utility in previous studies. S100A1 is a member of the calcium-binding protein family and has previously been identified to be upregulated in EOC compared to normal tissue and may act as a prognostic and therapeutic biomarker as it has shown to be associated with unfavorable prognosis when studying EC cell lines.[21] Contrary to these findings, here S100A1 was upregulated in HGSC and not among the strong prognostic proteins. VWA2 (EC) has recently been found to have predictive and prognostic potential in colorectal cancer, although the biological role of this extracellular protein is not fully understood.[22,23] Being extracellular and uniquely upregulated in EC, this protein may be a signature for this histotype while also being measurable in liquid biopsies. Serum levels of SPINK1 has predicted poor prognosis in several cancers, and was in this study the most upregulated protein in EC when compared to HGSC.[24] The highly abundant protein in HGSC compared to EC, SNCG, is a suggested therapeutic target in other cancers but has not been identified for EOC to date.[25] AGR2 (MC), a key protein in mucus-producing cells, has been linked to poor survival in EOC and is involved in the epithelial-mesenchymal transitioning, and has been shown to be a potential diagnostic biomarker for MC with overexpression in early stages.[26–28] Our study confirms the high abundance of AGR2 in MC and underlines the necessity to evaluate its diagnostic potential. In a previous study, CTH (CCC) was found to be of potential clinical importance for CCC carcinomas in EOC specifically with its capability of stratifying CCC and HGSC.[29] It is a critical component in transsulfuration, and most notably is evidenced to drive tumor progression in CCC.[30] These proteins, while having diagnostic potential contributed to their upregulation, may also be suitable for therapeutic targeting.

Figure 3. Gene set enrichment analysis for the full proteome and hallmark cancer pathways for the four main histotypes

(A) Heatmap illustrating overlap of the five most down- and upregulated enriched biological processes for respective histotype in gene set enrichment analysis. (B) Dot plots depicting the five uniquely down- and upregulated enriched BPs. (C) Dot plots displaying the most upregulated enriched cancer hallmark BPs. Significance was based on FDR threshold (<0.20), and then ranked from highest to lowest NES. BP, biological process; CCC, clear-cell ovarian carcinoma; EC, endometrioid ovarian carcinoma; FDR, false discovery rate; HGSC, high-grade serous ovarian carcinoma; MC, mucinous ovarian carcinoma; NES, normalized enrichment score.

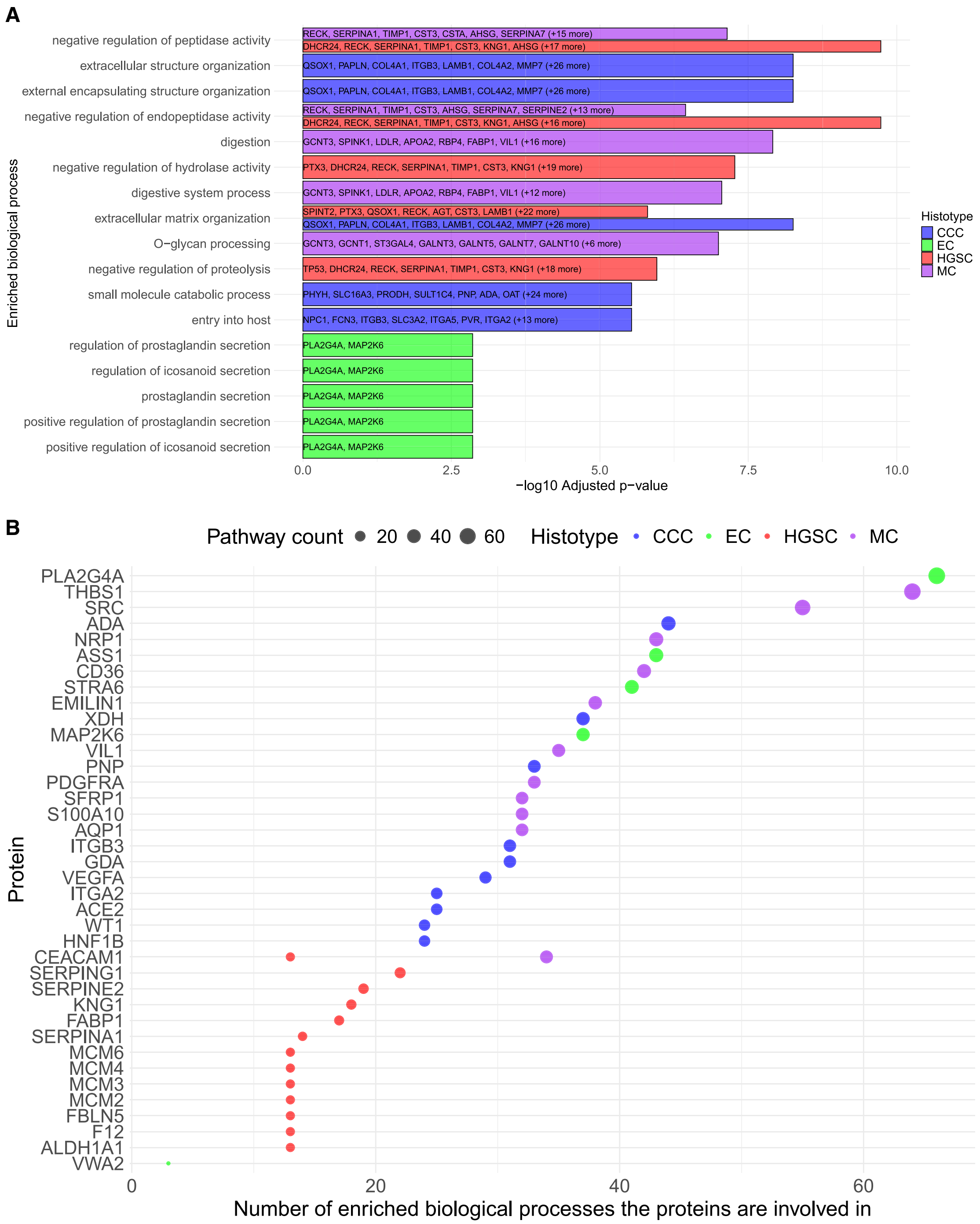

Figure 4. Gene ontology enrichment analysis based on differentially abundant proteins for the histotypes

(A) Dotplot of the five most significantly enriched (adjusted *p* value < 0.05) BPs for all histotypes along with associated DAPs for respective BP. *p* values were adjusted using false discovery rate. (B) Dotplot for the prevalence of the genes involved in all significantly enriched BPs for respective histotype. Adjusted *p* values were corrected for multiple testing using false discovery rate. BP, biological process; CCC, clear-cell ovarian carcinoma; EC, endometrioid ovarian carcinoma; HGSC, high-grade serous ovarian carcinoma; MC, mucinous ovarian carcinoma.

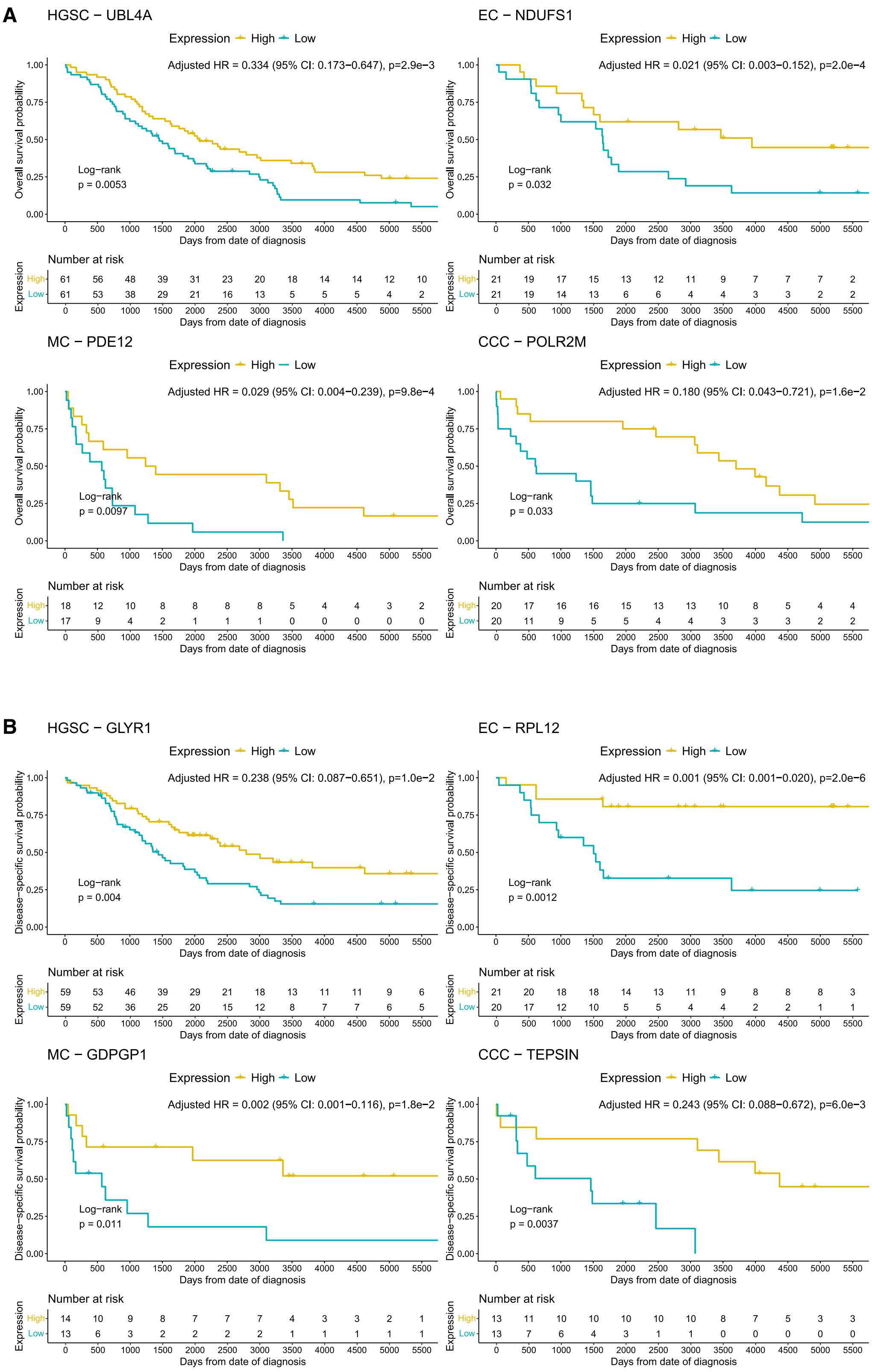

(legend on next page)

The DAPs identified by the pairwise comparisons may provide additional proteins for biomarker panels, especially considering that EC that displayed few DAPs when compared to the other histotypes. Additionally, proteins to distinguish between malignant, BL, and B may highlight key components driving disease development and progression. MX1 has been proposed as a therapeutic target in ovarian cancer as studies have identified its role in promoting cell migration and immune evasion.[31] In our study, MX1 was especially upregulated in HGSC when compared to SBL. Between SBL and B on the other hand, KRT23 was highly upregulated. In line with previous studies, KRT23 is overexpressed in EOC compared to normal tissue, and has been shown to regulate epithelial-mesenchymal transition.[32] The previously described protein S100A1 that was differentially abundant between HGSC and MC was also the most upregulated in SBL when compared to MBL. Deregulated proteins between MBL and SMBL can provide tools for differential diagnosis. AKR1B10 (upregulated in MBL) plays a key role in cancer via lipid metabolism, and has been shown to be a prognostic biomarker in breast and ovarian cancers.[33,34] More research is needed to stratify MBL and MB, as our study findings were inconclusive due to the relatively few samples for these subtypes.

The adopted SVM models on LASSO-selected DAPs generated proteins panels that may be used to stratify a histotype from the rest, but also for each pairwise comparison presented in this study, with high AUC scores. Notably, when identifying combinations of proteins to classify a histotype when compared to the others, only S100A1 was among the most up- or downregulated proteins (found in panel for HGSC). In the panels for the pairwise comparisons on the other hand, most of the DAPs of highest FC were included in the models such as AGR2 for HGSC vs. MC and VWA2 for HGSC vs. EC. This may be due to more heterogeneous abundance for the other histotypes the protein abundance is compared to, affecting the model performance during protein selection. Higher AUC scores for protein panels for BL and B tissues despite lower sample counts suggest more homogeneous protein abundances in or a result of model overfitting. Although our internal cross validation was robust, these models need to be tested on external data to confirm their generalizability.

The GSEA highlighted mutual and distinct histotype-specific enrichment of BPs when the effects of all expressed proteins were accounted for. The most significantly enriched processes based on NES revealed that MC and CCC shared downregulation of RNA splicing events via transesterification. Alterations in RNA splicing events is a common trait in the onset of tumorigenesis.[35] The downregulation of complement activation shared in HGSC and EC has underwent prognostic evaluation recently, where elevation of certain markers involved in this process has shown association with poorer OS in EOC patients.[36] Here, complement activation was highly upregulated in CCC. The high upregulation in HGSC and EC and downregulation in MC and CCC of DNA-templated replication and transcription further distinctly divided the histotypes. This process, when upregulated, induce genomic instability by replication stress, is a common feature for cancer.[37] Epithelial mesenchymal transition was upregulated in MC, which correlates with the high abundance of AGR2 in this histotype that has shown to be involved in this process.[27] The uniquely enriched pathways for respective histotype serve as potential BPs to further investigate histotype-specific traits. Mapping cancer hallmark BPs distinctly divided the histotypes into two common traits, where HGSC and EC were for example characterized by upregulation of E2F, MYC V1, and MYC V2 targets, whereas MC and CCC showed upregulation of complement.

GOEA focused on enrichment of BPs driven by the identified DAPs. The analysis identified large sets of proteins involved in the most significant enriched processes, and highlighted mutual and histotype-specific features. In contrast to the GSEA, HGSC and MC shared features, showing highly significant enrichment of negative regulation of peptidase and endopeptidase activity, a process that promotes angiogenesis and metastasis when dysregulated.[38] However, since GOEA does not provide directionality of the enrichment, the two histotypes may display opposite direction of regulation. EC distinguished itself from the other histotypes by high enrichment of prostaglandin and icosanoid secretion. Prostaglandins are known to be involved in tumor progression by regulation of PGE2, which is a protein that can enhance carcinogenesis and is known to induce chemoresistance in EOC.[39,40] Unique to CCC, extracellular structure and matrix organization were highly enriched. This is the regulation of key constituents involved in the interplay between cancer cells, and drives cell proliferation, migration, and apoptosis in cancer by alterations in the cellular matrix by regulation of collagen.[41,42] These processes could be utilized to investigate disease development and progression, and the involved proteins in such processes may be used as targets for therapies.

None of the DAPs involved in the most significantly enriched BPs in GOEA showed correlation with the most up- and downregulated DAPs, except SPINK1 for MC with its involvement in digestion and digestive system process. Instead, proteins from the serpin, MUC, and COL-family were common in HGSC, MC, and CCC, respectively. Observing the occurrence of proteins among all

Figure 5. Kaplan-Meier curves for proteins with the strongest association with favorable outcome

Kaplan-Meier curves for the survival probability for (A) lowest HR for all histotypes for OS and (B) DSS. Displayed p values for HR have been FDR-corrected. High and low strata groups are dichotomized based on median log2 intensity for each protein. Proteins were filtered for those passing significance (FDR <0.05) and bootstrap validation ($p < 0.20$) for 1,000 iterations of bootstrapped survival data. HR shown were estimated using multivariate cox regression, adjusted by stage, age, CA-125 levels, and residual tumor size at cytoreductive surgery after LASSO-selection of the covariates to retain non-zero contributions to the survival models. CCC, clear-cell ovarian carcinoma; DSS, disease-specific survival; EC, endometrioid ovarian carcinoma; FDR, false discovery rate; HGSC, high-grade serous ovarian carcinoma; HR, hazard ratio; LASSO, least absolute shrinkage and selection operator; MC, mucinous ovarian carcinoma; OS, overall survival.

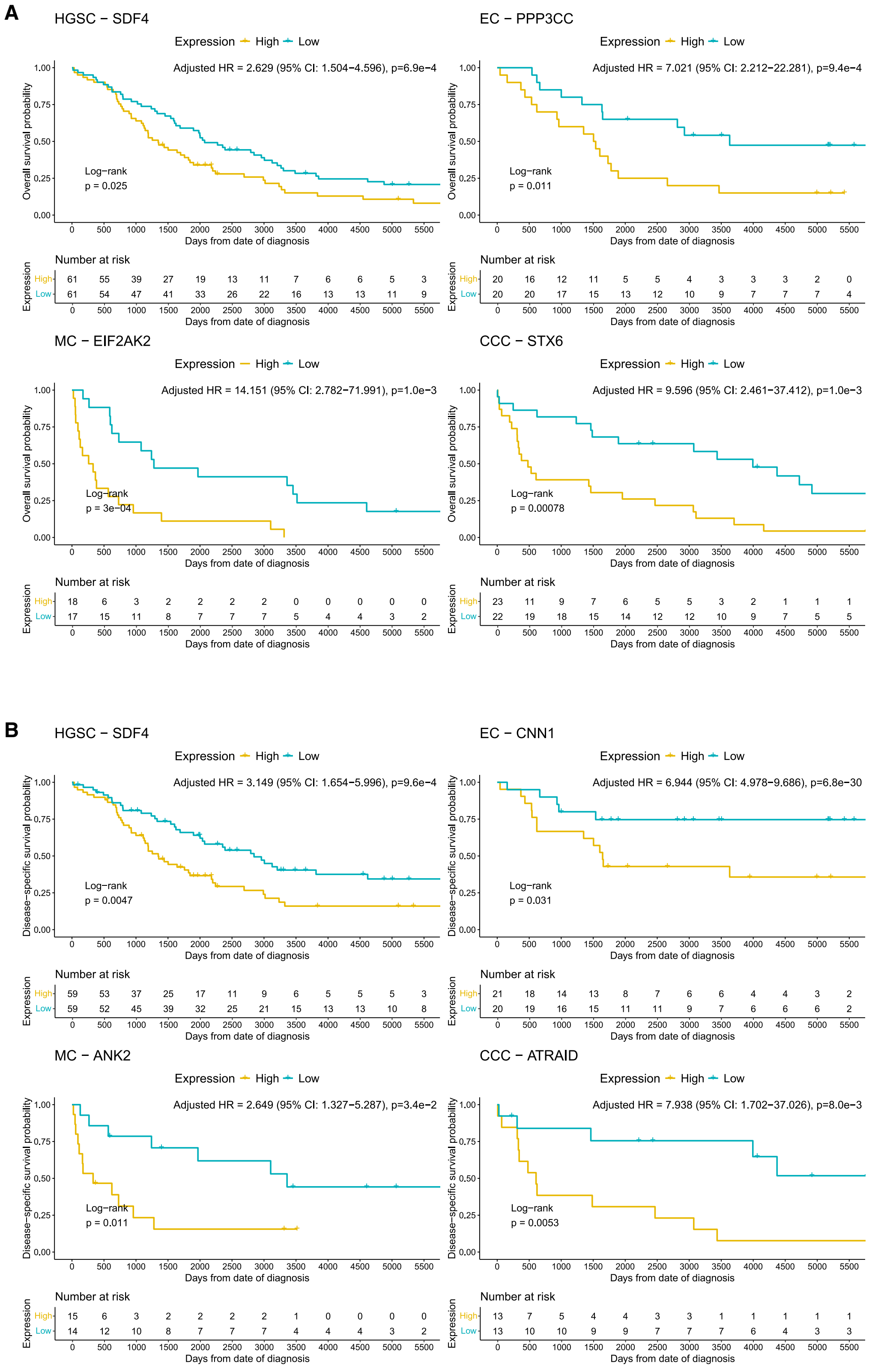

(legend on next page)

significantly enriched BPs, SERPING1, PLA2G4A, THBS1, and ADA emerged as the most recurrent protein for HGSC, EC, MC, and CCC, respectively. SERPING1 is a previously proposed biomarker candidate for stratification of HGSC with its elevated levels in tumor fluids compared to fluids from B tissue.[43] PLA2G4A has also showed association with EOC, as its higher abundance in a previous study indicates promotion of ovarian carcinogenesis.[44] THBS1 plays an active role in suppressing cancer development by preventing angiogenesis, and was in this study upregulated in MC.[45] The isoenzymes of ADA, namely ADA1 and ADA2, have been suggested to have elevated levels in HGSC and ADA2 association with favorable prognosis in this histotype.[46]

Survival analysis identified histotype-specific prognostic proteins for OS and DSS, which may be used to expand the current repertoire of suggested prognostic biomarkers and act as therapeutic targets. For favorable survival, the proteasome delivery and tumor suppressing protein UBLA4 has previously shown favorable prognosis in other cancer types and was favorable for HGSC (OS) in the present study.[47,48] GLYR1, the favorable protein for HGSC (DSS), is a transcription regulator having shown favorable prognosis in colorectal cancer.[49,50] For EC, the favorable proteins NDUFS1 (OS) and RPL12 (DSS) are mitochondrial constituents. NDUFS1 (OS) is thought to be associated with malignant transformation in endometriosis, whereas the role of RPL12 warrants more research.[51] Although the polymerase subunit POLR2M showed favorable prognosis (OS) for CCC in our cohort, it has been found to be associated with poor prognosis in acute myeloid leukemia by silencing the MIR139 tumor suppressor.[52] For MC, top candidates for improved survival need to be investigated further as research is lacking (PDE12 for OS, GDPGP1 for DSS). As most of the favorable proteins have not been associated with EOC previously, they may serve as additional prognostic tools.

Proteins involved in increased risk of death have the potential to be used for multiple clinical purposes beyond prognosis and therapeutic intervention. The calcium-binding protein SDF4 is secreted from solid tumors and could therefore be used for diagnostic and therapeutic purposes, and was the most unfavorable protein in HGSC (OS and DSS).[53] PPP3CC displayed the highest risk of death (OS) in EC, but this modulator of the phosphorylation of transcription factors has previously been shown to promote favorable prognosis in EOC, but not EC specifically which suggests it may be a suitable target for this histotype.[54] PPP3CC in EOC therefore needs to be investigated further. EIF2AK2 also presented conflicting results. Activation of this translation initiation kinase suppresses protein translation and is also evidenced to induce chemo-sensitive properties in EOC.[55] Here, however, unfavorable prognosis was found for MC (OS). Consistent with previous studies on the other hand, the poor prognosis (OS) associated with high STX6 expression has previously shown poorer survival in EOC. This is a protein that regulates transportation at the surface of the Golgi apparatus.[56] For DSS, biomarker candidates for unfavorable prognosis for EC (CNN1) and the key cell development protein ANK2 (MC) have presented oncogenic properties in EOC and other cancers, respectively. These candidates should be subjected to targeted studies, whereas the prognostic role for the membrane adapter protein ATRAID (CCC) needs further investigation.[57–59]

This study has several limitations. Sample sizes for certain subtypes such as LGSC ($n = 8$) and SMBL ($n = 4$) were too low for statistical analysis, introducing uncertainty and limiting power. Hence, LGSC was excluded, and results where SMBL is included may be unreliable. Survival data were limited for MC and CCC, particularly for DSS, reducing the number of proteins passing bootstrap validation and increasing the risk of overfitted Cox models. While the identified candidates remain promising, a larger sample size and more complete clinical data might have revealed additional prognostic proteins. Thus, further investigation with more patient samples and external validation are needed. Additionally, patients were diagnosed between the 1990s and 2020s, during which diagnostic and treatment advances occurred as well as centralization of ovarian cancer care (2011) which is potentially biasing survival analyses, although this has been indirectly accounted for by using completeness of cytoreductive surgery as a covariate. Accounting for treatment regimens may improve identification of robust prognostic markers.

In conclusion, we identified histotype-specific proteins differentially expressed between EOC histotypes and BL/B tumors, forming biomarker panels that may aid tissue stratification. Enriched biological processes (BPs) were profiled for each histotype, revealing DAPs linked to histotype-specific dysregulation. Prognostic protein panels were found for each histotype, associated with overall- and disease-specific survival. The high abundance of proteins such as S100A1, AGR2, CTH, and KRT23 correlated well with previous studies on ovarian tissues. On the other hand, much less is known about the association with EOC for the identified prognostic biomarkers, although most of them have been found to be of prognostic or therapeutic value in other malignancies. This study highlights both known and unexplored diagnostic and prognostic biomarkers, proposing clinical tools for EOC with histotype-specificity and ability to distinguish tissue types. However, the biological roles of these

Figure 6. Kaplan-Meier curves for proteins with the strongest association with unfavorable outcome

Kaplan-Meier curves for the survival probability for (A) highest HR for all histotypes for OS and (B) DSS. Displayed p values for HR have been FDR-corrected using the Benjamini-Hochberg model. High and low strata groups are dichotomized based on median log2 intensity for each protein. Proteins were filtered for those passing significance (FDR <0.05) and bootstrap validation ($p < 0.20$) for 1,000 iterations of bootstrapped survival data. HR shown were estimated using multivariate cox regression, adjusted by stage, age, CA-125 levels, and residual tumor size at cytoreductive surgery after LASSO-selection of the covariates to retain non-zero contributions to the survival models. CCC, clear-cell ovarian carcinoma; DSS, disease-specific survival; EC, endometrioid ovarian carcinoma; FDR, false discovery rate; HGSC, high-grade serous ovarian carcinoma; HR, hazard ratio; LASSO, least absolute shrinkage and selection operator; MC, mucinous ovarian carcinoma; OS, overall survival.

proteins require further investigation, and their clinical utility ought to be validated using external data and immunohistochemistry, particularly in the context of complementarity to established histopathological diagnosis for EOC histotypes. Additional studies on enriched BPs and DAP functions could further clarify the mechanisms underlying disease progression.

MATERIALS AND METHODS

Patients and tumor samples

Primary invasive epithelial ovarian carcinomas from 252 patients diagnosed between 1993 and 2022, as well as 17 MB, serous, cystadenoma, and cystadenofibroma, and 31 BL tumors of serous, MC, seromucinous and EC type diagnosed between 2007 and 2015 were obtained from the fresh-frozen tumor bank at the Sahlgrenska University Hospital Oncology lab (Gothenburg, Sweden). Low-grade serous ovarian carcinoma was excluded from the study due to low sample counts. Clinicopathologic and survival data were obtained from the National Quality Registry at the Regional Cancer Center West (Gothenburg, Sweden) and the Cancer Registry at the National Board of Health and Welfare, respectively. The study cohort was compiled according to the International Federation of Gynecology and Obstetrics (FIGO) stages I, II, III, and IV, and the survival data were calculated from date of initial diagnosis to the date of death of any cause for overall survival or death from EOC for disease-specific survival.

Tumor specimens were reclassified by a board-certified pathologist at Sahlgrenska University Hospital using the 2020 WHO criteria regarding histotype and histological grade. Four micrometer full-faced sections from formalin-fixed paraffin-embedded (FFPE) blocks were used when available and cryosections if no FFPE block was available and stained with hematoxylin and eosin in either case. All procedures were performed in accordance with the Declaration of Helsinki and approved by the Regional Ethical Review Board (Gothenburg, Sweden; case numbers 767-14 and 201-15, and complementary case numbers T973–15 and T333-16). The Regional Ethical Review Board approved a waiver of written consent to use the tumor specimens.

Sample run with liquid chromatography-mass spectrometry and identification and quantification

Lysation of cut tissue pieces was performed with a Covaris ML230 ultrasonicator in 2% sodium dodecyl sulfate and 50 mM triethylammonium bicarbonate (TEAB). A Pierce BCA Protein Assay Kit (Thermo Fisher Scientific) was used to estimate protein concentration in the resulting lysates. Fifty microgram protein aliquots were reduced in 10 mM dithiothreitol at 56°C for 30 min and then alkylated in 20 mM chloroacetamide at room temperature for 10 min. Protein samples were added to washed hydrophobic and hydrophilic Sera-Mag SpeedBeads (Carboxylate-Modified, Cytiva) in a 10:1 bead-to-protein ratio. The SP3-workflow was adapted from the protein and peptide clean-up for mass spectrometry protocol.[60] Proteins were precipitated on the beads by ethanol and then washed and dried at room temperature. Proteins were digested with 1 μg LysC + trypsin (Promega and Thermo Fisher Scientific, respectively) in 50 mM TEAB by incubating at 37°C overnight while shaking. An additional 1 μg of trypsin was added and digested for three hours. Resulting peptides were purified on the beads, eluted and concentration was determined using the Pierce Quantitative Peptide Assay (Thermo Fisher Scientific). Estimated 400 ng of peptides was dissolved in 0.1% formic acid (FA) and 0.15% n-dodecyl-beta-D-maltoside (DDM) and was loaded onto Evotips Pure (Evosep) according to the manufacturers' instructions.

The peptides were analyzed on a timsTOF HT mass spectrometer (Bruker) coupled to an Evosep One liquid chromatography (LC) system (Evosep) with a Pepsep C18 column (15 cm × 150 μm ID, 1.5 μm particle size). The LC system ran the 30 samples per day (30SPD) method. The timsTOF was run in DIA-PASEF mode with 10 PASEF/MSMS scans. The samples were matched using directDIA (Swissprot, June 2023, 20407 entries) with Spectronaut (v. 18.6.231227.55695). Strict trypsin with 1 missed cleavage for protein digestion was set. Methionine oxidation and N-terminus acetylation were set as variable modifications, and carbamidomethylation of cysteine was set as fixed modification. The retention time and ion-mobility value were automatically selected and only b- and y-ions were used. Default settings were used for identification and matching toward directDIA spectra libraries and quantification was performed on Only Protein Group Specific.

Data pre-processing and analysis environment

Label-free cross normalization was performed using local normalization with no filter type while running the raw MS1 and MS2 data in directDIA in Spectronaut (v. 18.6.231227.55695). The imported annotations from the directDIA mode were PG. ProteinGroups, PG.Genes, and PG.ProteinDescriptions. MS2 intensities were used as abundances for the proteins in all subsequent processing steps and analyses. Data were filtered to retain proteins that had quantitative values in over 70% of the samples for at least one condition (histotype herein). Filtered data were then log2-transformed. All subsequent data pre-processing and analysis were performed in R (v. 4.3.3), apart from directDIA for protein searching.

Differential protein abundance and statistical analysis

DA analysis was done by performing unpaired limma t tests using the NormalyzerDE package (v. 1.20.0), where two-sided adjusted p values using FDR correction were generated as well as log2 FCs.[61] Proteins were considered significantly up- or downregulated at an FDR <0.05 and FC greater than 1.5 for upregulation and lower than −1.5 for downregulation. Two different sets of most DAPs between histotypes were generated. In one set, significant proteins for each histotype in pairwise comparisons were obtained, including comparisons with BL and B tissues. In the other set, the abundance of proteins for a histotype was compared to all histotypes at the same time by labeling the others as one combined histotype to acquire FDR and log2 FCs for a histotype compared to the others. Pairwise comparisons between the log2 intensities of the proteins between

histotypes were made with the Wilcoxon test and p values were adjusted for multiple testing using FDR for the boxplots.

Identifying biomarker panels for all comparisons

Logistic regression with LASSO using elastic net (alpha = 0.5) was used to pre-select DAPs for a histotype using glmnet (v. 4.1–8). Features (proteins) were selected using lambda min with 5-fold cross-validation and classification based on AUC, filtering for DAPs with non-zero coefficients. Filtered proteins were then used to train support vector machine (SVM) models using kernlab (v. 0.9–33) to find the combination of proteins with the highest AUC score for distinguishing a histotype from the others and for pairwise comparisons. SVM models split the protein abundance data into an 80% training set, and 20% testing set to evaluate the classification performance with confusion matrices with for sensitivity and specificity, AUC scores and receiver operating characteristics (ROC) curves. SVM modeling was employed by first identifying the LASSO-selected protein of highest AUC score. Then, the other pre-selected proteins were iteratively added to the model with the best performing protein, only keeping the protein in the model if the AUC score increased. One protein at a time was added to the SVM model until there were no more proteins that improved the performance. Then, additional proteins were added to the model until the AUC score decreased 1% point below the maximum score achieved to increase the size of the protein panels without penalizing performance, capping to a maximum of five proteins included in the model.

Gene set enrichment analysis

The full proteome was used to feed into the GSEA. For each histotype, the proteins were ranked by the log2 FCs for a histotype when compared to all other histotypes combined, ordered from highest to lowest log2 FC. GSEA was performed for each histotype independently using the gseGO-function in clusterProfiler (v. 4.10.1). The results were filtered with a Benjamini-Hochberg-adjusted p value cutoff of 0.05 and estimated normalized enrichment scores (NES) were used to create dot plots with the five most upregulated and five most downregulated biological processes based on the scores along with their respective counts between a given histotype and the other histotypes. The analysis was repeated with imported cancer hallmark libraries to generate significance and NES for these pathways.

Gene ontology enrichment analysis

The DAPs for each histotype from the DEA were used to perform gene ontology (GO) enrichment analysis. The same workflow and significance cutoff for adjusted p-value at 0.05 as for GSEA was adapted, with the modification of using the enrichGO-function for the enrichment analysis from clusterProfiler (v. 4.10.1). The top 5 GO-terms based on significance were filtered and used for barplots.

Survival analysis and validation of robustness of models

Obtaining histotype-specific proteins associated with overall survival and disease-specific survival was performed by generating Cox proportional hazards models using coxph from the survival package (v. 3.7–0). For each histotype independently, univariate Cox regression was iterated over the full proteome to generate lists of all proteins associated with survival based on Wald test p values ($p < 0.05$) along with their respective HRs, confidence intervals (CI), and C-index. Proteins filtered for significance were subjected to a log rank test to filter for proteins showing significant log rank p value ($p < 0.05$) when dichotomizing data into high and low expression using the median expression of the protein as threshold. Proteins fulfilling these criteria then underwent multivariate Cox regression on variables selected by LASSO to have a non-zero coefficient. The model for each protein was adjusted for age, CA-125 levels, stage, and residual tumor size at cytoreductive surgery. Survival model robustness was assessed with 1,000 bootstrap iterations, extracting the average p value. A significance threshold of bootstrap p value <0.2 was used. Proteins were divided into proteins associated with decreased risk (HR < 1) and increased risk (HR > 1) of death (CI not spanning 1). Kaplan-Meier curves were plotted with data dichotomized by the same protocol as for the log rank test.

DATA AND CODE AVAILABILITY

The mass spectrometry and Spectronaut analysis files have been deposited to the MassIVE repository with the dataset identifier MSV000097588 and is publicly available as of the date of publication. All analysis code is available at https://github.com/lwernerGU/Proteomics-analysis-pipeline.git and is publicly available as of the date of publication.

ACKNOWLEDGMENTS

This project has been supported by grants from the Swedish Cancer Society (23 2732 Pj 01 H), King Gustav V Jubilee Clinic Cancer Research Foundation (2022:410), the LUA/ALF-agreement in West of Sweden health care region (ALFGBG-1005885), Herbert & Karin Jacobssons Stiftelse, Assar Gabrielsson Research Foundation for Clinical Cancer Research (FB 23-47), and Sahlgrenska University Hospital Research Foundations and Lions Cancer Research Fund of Western Sweden (2024:20). We would like to thank Johannes Fuchs at Proteomics Core Facilities, University of Gothenburg for performing library search with the raw MS-data and Shahin De Lara, BMA, at Sahlgrenska University Hospital for the FFPE collection. We would also like to thank Karin Sundfeldt, Professor, MD, at the Department of Obstetrics and Gynecology, University of Gothenburg, for providing patient samples. We are also grateful to Daniella Pettersson, research assistant, at the Department of Clinical Pathology, Sahlgrenska University Hospital, for aiding in sectioning FFPE material.

AUTHOR CONTRIBUTIONS

K.H. was responsible for the outline of the study concept, retrieval of clinical data, and together with T.Z.P., L.W., P.D.-K., P.K., E.F.-A, and G.S. the experimental design and study group selection. H.S. and E.I. contributed with the collection of FFPE material for the patients. A.K. and E.W.R. selected suitable primary tumor blocks and evaluated fresh-frozen imprints and tumor regions of H&E-stained FFPE material. C.M., E.W. R., and A.K. performed the pathological reclassification of the histotypes. A.T. was the mainly responsible for the planning of the experimental setup for the LC-MS runs and generating the raw mass spectrometry data and E.R. was responsible for executing the practical work. E.I., H.S., F.L., P.E., and A.S.T. aided with bioinformatical support. L. W. performed all practical experiments apart from LC-MS sample preparation and running, wrote all R scripts, analyzed the data, created the figures, tables, and wrote the full manuscript. All authors have reviewed, edited, and approved the final manuscript.

DECLARATION OF INTERESTS

The authors declare no competing interests.

SUPPLEMENTAL INFORMATION

Supplemental information can be found online at https://doi.org/10.1016/j.omton.2025.201019.

REFERENCES

1. McGregor, S.M. (2022). Pathologic Classification of Ovarian Cancer. Methods Mol. Biol. *2424*, 11–40.
2. Pullen, R.L., Jr. (2024). Ovarian cancer. Nursing *54*, 17–28.
3. Sideris, M., Menon, U., and Manchanda, R. (2024). Screening and prevention of ovarian cancer. Med. J. Aust. *220*, 264–274.
4. Köbel, M., and Kang, E.Y. (2022). The evolution of ovarian carcinoma subclassification. Cancers *14*, 416. https://doi.org/10.3390/cancers14020416.
5. Kuroki, L., and Guntupalli, S.R. (2020). Treatment of epithelial ovarian cancer. BMJ *371*, m3773. https://doi.org/10.1136/bmj.m3773.
6. Zhang, M., Cheng, S., Jin, Y., Zhao, Y., and Wang, Y. (2021). Roles of CA125 in diagnosis, prediction, and oncogenesis of ovarian cancer. Biochim. Biophys. Acta. Rev. Cancer *1875*, 188503. https://doi.org/10.1016/j.bbcan.2021.188503.
7. Bonifácio, V.D. (2020). Ovarian cancer biomarkers: moving forward in early detection. Adv. Exp. Med. Biol. *1219*, 355–363. https://doi.org/10.1007/978-3-030-34025-4_18.
8. Björkman, K., Mustonen, H., Kaprio, T., Kekki, H., Pettersson, K., Haglund, C., and Böckelman, C. (2021). CA125: A superior prognostic biomarker for colorectal cancer compared to CEA, CA19-9 or CA242. Tumour. Biol. *43*, 57–70. https://doi.org/10.3233/TUB-200069.
9. Zhang, R., Siu, M.K.Y., Ngan, H.Y.S., and Chan, K.K.L. (2022). Molecular biomarkers for the early detection of ovarian cancer. Int. J. Mol. Sci. *23*, 12041. https://doi.org/10.3390/ijms231912041.
10. Stewart, C., Ralyea, C., and Lockwood, S. (2019). Ovarian cancer: an integrated review. In Seminars in Oncology Nursing (Elsevier).
11. Muinao, T., Deka Boruah, H.P., and Pal, M. (2018). Diagnostic and Prognostic Biomarkers in ovarian cancer and the potential roles of cancer stem cells–An updated review. Exp. Cell Res. *362*, 1–10.
12. Stany, M.P., Bonome, T., Wamunyokoli, F., Zorn, K., Ozbun, L., Park, D.-C., Hao, K., Boyd, J., Sood, A.K., Gershenson, D.M., et al. (2008). Classification of ovarian cancer: a genomic analysis. Adv. Exp. Med. Biol. *622*, 23–33. https://doi.org/10.1007/978-0-387-68969-2_3.
13. Kurnit, K.C., Fleming, G.F., and Lengyel, E. (2021). Updates and new options in advanced epithelial ovarian cancer treatment. Obstet. Gynecol. *137*, 108–121. https://doi.org/10.1097/AOG.0000000000004173.
14. Cancerfonden. Cancer i siffror 2023.
15. Dhar, C., Ramachandran, P., Xu, G., Pickering, C., Čaval, T., Wong, M., Rice, R., Zhou, B., Srinivasan, A., Aiyetan, P., et al. (2024). Diagnosing and staging epithelial ovarian cancer by serum glycoproteomic profiling. Br. J. Cancer *130*, 1716–1724. https://doi.org/10.1038/s41416-024-02644-4.
16. Mukherjee, S., Sundfeldt, K., Borrebaeck, C.A.K., and Jakobsson, M.E. (2021). Comprehending the proteomic landscape of ovarian cancer: a road to the discovery of disease biomarkers. Proteomes *9*, 25. https://doi.org/10.3390/proteomes9020025.
17. Qian, L., Zhu, J., Xue, Z., Zhou, Y., Xiang, N., Xu, H., Sun, R., Gong, W., Cai, X., Sun, L., et al. (2024). Proteomic landscape of epithelial ovarian cancer. Nat. Commun. *15*, 6462.
18. Gong, T.-T., Guo, S., Liu, F.-H., Huo, Y.-L., Zhang, M., Yan, S., Zhou, H.-X., Pan, X., Wang, X.-Y., Xu, H.-L., et al. (2023). Proteomic characterization of epithelial ovarian cancer delineates molecular signatures and therapeutic targets in distinct histological subtypes. Nat. Commun. *14*, 7802.
19. Subramanian, A., Tamayo, P., Mootha, V.K., Mukherjee, S., Ebert, B.L., Gillette, M. A., Paulovich, A., Pomeroy, S.L., Golub, T.R., Lander, E.S., and Mesirov, J.P. (2005). Gene set enrichment analysis: a knowledge-based approach for interpreting genome-wide expression profiles. Proc. Natl. Acad. Sci. USA *102*, 15545–15550.
20. Liberzon, A., Birger, C., Thorvaldsdóttir, H., Ghandi, M., Mesirov, J.P., and Tamayo, P. (2015). The molecular signatures database hallmark gene set collection. Cell Syst. *1*, 417–425.
21. Jin, W., Hui, H., Jiang, J., Li, B., Deng, Z., and Tuo, X. (2024). S100A1 overexpression stimulates cell proliferation and is predictive of poor outcome in ovarian cancer. Transl. Cancer Res. *13*, 5265–5277.
22. Tian, Y., Zhao, Q., Wu, H., Guo, J., and Wu, H. (2024). VWA2 protein molecular mechanism predicts colorectal cancer: Promoting cell invasion and migration by inhibiting NK cell activation. Int. J. Biol. Macromol. *279*, 135394.
23. González, B., Fece de la Cruz, F., Samuelsson, J.K., Alibés, A., and Alonso, S. (2018). Epigenetic and transcriptional dysregulation of VWA2 associated with a MYC-driven oncogenic program in colorectal cancer. Sci. Rep. *8*, 11097.
24. Räsänen, K., Itkonen, O., Koistinen, H., and Stenman, U.-H. (2016). Emerging roles of SPINK1 in cancer. Clin. Chem. *62*, 449–457.
25. Liu, J., Shao, T., Zhang, J., Liu, Q., Hua, H., Zhang, H., Wang, J., Luo, T., Shi, Y.E., and Jiang, Y. (2022). Gamma synuclein promotes cancer metastasis through the MKK3/6-p38MAPK cascade. Int. J. Biol. Sci. *18*, 3167–3177.
26. Ly, T.T.G., Yun, J., Ha, J.-S., Kim, Y.-J., Jang, W.-B., Van Le, T.H., Rethineswaran, V. K., Choi, J., Kim, J.-H., Min, S.-H., et al. (2022). Inhibitory effect of Etravirine, a non-nucleoside reverse transcriptase inhibitor, via anterior gradient protein 2 homolog degradation against ovarian cancer metastasis. Int. J. Mol. Sci. *23*, 944.
27. Martisova, A., Sommerova, L., Krejci, A., Selingerova, I., Kolarova, T., Zavadil Kokas, F., Holanek, M., Podhorec, J., Kazda, T., and Hrstka, R. (2022). Identification of AGR2 gene-specific expression patterns associated with epithelial-mesenchymal transition. Int. J. Mol. Sci. *23*, 10845.
28. Park, K., Chung, Y.J., So, H., Kim, K., Park, J., Oh, M., Jo, M., Choi, K., Lee, E.-J., Choi, Y.-L., et al. (2011). AGR2, a mucinous ovarian cancer marker, promotes cell proliferation and migration. Exp. Mol. Med. *43*, 91–100.
29. Hughes, C.S., McConechy, M.K., Cochrane, D.R., Nazeran, T., Karnezis, A.N., Huntsman, D.G., and Morin, G.B. (2016). Quantitative profiling of single formalin fixed tumour sections: proteomics for translational research. Sci. Rep. *6*, 34949.
30. EL-Naggar, A.M., Li, Y., Turgu, B., Ding, Y., Wei, L., Chen, S.Y., Trigo-Gonzalez, G., Kalantari, F., Vallejos, R., Lynch, B., et al. (2025). Cystathionine gamma-lyase-mediated hypoxia inducible factor 1-alpha expression drives clear cell ovarian cancer progression. J. Pathol. *266*, 352–367.
31. Chang, H., Zhu, Y., Zheng, J., Chen, L., Lin, J., and Yao, J. (2021). Construction of a Macrophage Infiltration Regulatory Network and Related Prognostic Model of High-Grade Serous Ovarian Cancer. J. Oncol. *2021*, 1331031.
32. Ren, M., Gao, Y., Chen, Q., Zhao, H., Zhao, X., and Yue, W. (2020). The overexpression of keratin 23 promotes migration of ovarian cancer via epithelial-mesenchymal transition. BioMed Res. Int. *2020*, 8218735.
33. Qu, J., Li, J., Zhang, Y., He, R., Liu, X., Gong, K., Duan, L., Luo, W., Hu, Z., Wang, G., et al. (2021). AKR1B10 promotes breast cancer cell proliferation and migration via the PI3K/AKT/NF-κB signaling pathway. Cell Biosci. *11*, 163.
34. Yuan, D., Zhu, H., Wang, T., Zhang, Y., Zheng, X., and Qu, Y. (2023). Development and validation of an individualized gene expression-based signature to predict overall survival of patients with high-grade serous ovarian carcinoma. Eur. J. Med. Res. *28*, 465.
35. Zhang, Y., Qian, J., Gu, C., and Yang, Y. (2021). Alternative splicing and cancer: a systematic review. Signal Transduct. Target. Ther. *6*, 78.
36. Ricciuti, J., Liu, Q., Khan, A.N.M.N.H., Joseph, J.M., Veuskens, B., Giridharan, T., Suzuki, S., Emmons, T., Yaffe, M., Kuijpers, T.W., et al. (2025). Prognostic significance of serum complement activation, neutrophil extracellular traps and extracellular DNA in newly diagnosed epithelial ovarian cancer. Gynecol. Oncol. *193*, 49–57.
37. Gaillard, H., García-Muse, T., and Aguilera, A. (2015). Replication stress and cancer. Nat. Rev. Cancer *15*, 276–289.
38. Kos, J. (2022). Proteases: role and function in cancer. Int. J. Mol. Sci. *23*, 4632.
39. Jara-Gutiérrez, Á., and Baladrón, V. (2021). The role of prostaglandins in different types of cancer. Cells *10*, 1487.
40. Wang, Q., Morris, R.J., Bode, A.M., and Zhang, T. (2022). Prostaglandin pathways: opportunities for cancer prevention and therapy. Cancer Res. *82*, 949–965.
41. Huang, J., Zhang, L., Wan, D., Zhou, L., Zheng, S., Lin, S., and Qiao, Y. (2021). Extracellular matrix and its therapeutic potential for cancer treatment. Signal Transduct. Target. Ther. *6*, 153.

42. Walker, C., Mojares, E., and del Río Hernández, A. (2018). Role of extracellular matrix in development and cancer progression. Int. J. Mol. Sci. *19*, 3028.

43. Poersch, A., Grassi, M.L., Carvalho, V.P.d., Lanfredi, G.P., Palma, C.d.S., Greene, L. J., de Sousa, C.B., Carrara, H.H.A., Candido Dos Reis, F.J., and Faça, V.M. (2016). A proteomic signature of ovarian cancer tumor fluid identified by highthroughput and verified by targeted proteomics. J. Proteomics *145*, 226–236.

44. Zhu, L., Zhang, H., Zhang, X., Chen, R., and Xia, L. (2024). Drug repositioning and ovarian cancer, a study based on Mendelian randomisation analysis. Front. Oncol. *14*, 1376515.

45. Zeng, Z., Lin, C., Zhang, M.C., Kossinna, P., Wang, P., Cao, D., Wang, J., Xu, M., Wang, X., Li, Q., et al. (2023). Enterolactone and trabectedin suppress epithelial ovarian cancer synergistically via upregulating THBS1. Phytother Res. *37*, 4722–4739.

46. Gao, Z.W., Yang, L., Liu, C., Wang, X., Guo, W.T., Zhang, H.Z., and Dong, K. (2022). Distinct Roles of Adenosine Deaminase Isoenzymes ADA1 and ADA2: A Pan-Cancer Analysis. Front. Immunol. *13*, 903461.

47. Hessa, T., Sharma, A., Mariappan, M., Eshleman, H.D., Gutierrez, E., and Hegde, R. S. (2011). Protein targeting and degradation are coupled for elimination of mislocalized proteins. Nature *475*, 394–397.

48. Chen, H., Li, L., Hu, J., Zhao, Z., Ji, L., Cheng, C., Zhang, G., Zhang, T., Li, Y., Chen, H., et al. (2019). UBL4A inhibits autophagy-mediated proliferation and metastasis of pancreatic ductal adenocarcinoma via targeting LAMP1. J. Exp. Clin. Cancer Res. *38*, 297.

49. Fei, J., Ishii, H., Hoeksema, M.A., Meitinger, F., Kassavetis, G.A., Glass, C.K., Ren, B., and Kadonaga, J.T. (2018). NDF, a nucleosome-destabilizing factor that facilitates transcription through nucleosomes. Genes Dev. *32*, 682–694.

50. Hu, Z., Long, T., Ma, Y., Zhu, J., Gao, L., Zhong, Y., Wang, X., Wang, X., and Li, Z. (2020). Downregulation of GLYR1 contributes to microsatellite instability colorectal cancer by targeting p21 via the p38MAPK and PI3K/AKT pathways. J. Exp. Clin. Cancer Res. *39*, 76.

51. Ren, F., Wang, D.-B., Li, T., Chen, Y.-H., and Li, Y. (2014). Identification of differentially methylated genes in the malignant transformation of ovarian endometriosis. J. Ovarian Res. *7*, 73.

52. Stavast, C.J., van Zuijen, I., Karkoulia, E., Özçelik, A., van Hoven-Beijen, A., Leon, L. G., Voerman, J.S.A., Janssen, G.M.C., van Veelen, P.A., Burocziova, M., et al. (2022). The tumor suppressor MIR139 is silenced by POLR2M to promote AML oncogenesis. Leukemia *36*, 687–700.

53. Shinozuka, T., Kanda, M., Shimizu, D., Umeda, S., Takami, H., Inokawa, Y., Hattori, N., Hayashi, M., Tanaka, C., Nakayama, G., and Kodera, Y. (2023). Identification of stromal cell-derived factor 4 as a liquid biopsy-based diagnostic marker in solid cancers. Sci. Rep. *13*, 15540.

54. Anastasiadou, E., Messina, E., Sanavia, T., Labruna, V., Ceccarelli, S., Megiorni, F., Gerini, G., Pontecorvi, P., Camero, S., Perniola, G., et al. (2021). Calcineurin gamma catalytic subunit PPP3CC inhibition by miR-200c-3p affects apoptosis in epithelial ovarian cancer. Genes *12*, 1400.

55. Yin, L., Zeng, Y., Zeng, R., Chen, Y., Wang, T.-L., Rodabaugh, K.J., Yu, F., Natarajan, A., Karpf, A.R., and Dong, J. (2021). Protein kinase RNA-activated controls mitotic progression and determines paclitaxel chemosensitivity through B-cell lymphoma 2 in ovarian cancer. Oncogene *40*, 6772–6785.

56. Zhang, W., Lv, Q., Lu, D., and Chen, F. (2023). Syntaxin 6 enhances the progression of epithelial ovarian cancer by promoting cancer cell proliferation. Asian Pac. J. Cancer Prev. *24*, 2003–2010.

57. Yamane, T., Asanoma, K., Kobayashi, H., Liu, G., Yagi, H., Ohgami, T., Ichinoe, A., Sonoda, K., Wake, N., and Kato, K. (2015). Identification of the critical site of calponin 1 for suppression of ovarian cancer properties. Anticancer Res. *35*, 5993–5999.

58. Tian, Y., Xie, T., and Sun, X. (2022). Analysis of the regulatory mechanisms of prognostic immune factors in thyroid cancer. Front. Oncol. *12*, 1059591.

59. Mogal, M.R., Mahmod, M.R., Sompa, S.A., Junayed, A., Abedin, M.Z., and Sikder, M.A. (2021). Association between ankyrin 2 gene and breast cancer progression: A preliminary computational assessment using the database approach. Inform. Med. Unlocked *25*, 100663.

60. Hughes, C.S., Foehr, S., Garfield, D.A., Furlong, E.E., Steinmetz, L.M., and Krijgsveld, J. (2014). Ultrasensitive proteome analysis using paramagnetic bead technology. Mol. Syst. Biol. *10*, 757.

61. Willforss, J., Chawade, A., and Levander, F. (2019). NormalyzerDE: online tool for improved normalization of omics expression data and high-sensitivity differential expression analysis. J. Proteome Res. *18*, 732–740.

OMTON, Volume *33*

Supplemental information

Unveiling histotype-specific biomarkers in ovarian carcinoma using proteomics

Lucas Werner, Ella Ittner, Hugo Swenson, Elisabeth Werner Rönnerman, Claudia Mateoiu, Anikó Kovács, Pernilla Dahm-Kähler, Per Karlsson, Annika Thorsell, Elham Rekabdar, Parisa Esmaeili, Fredrik Levander, Eva Forssell-Aronsson, Axel Stenmark Tullberg, Ghassan Saed, Toshima Z. Parris, and Khalil Helou

Supplemental Figures

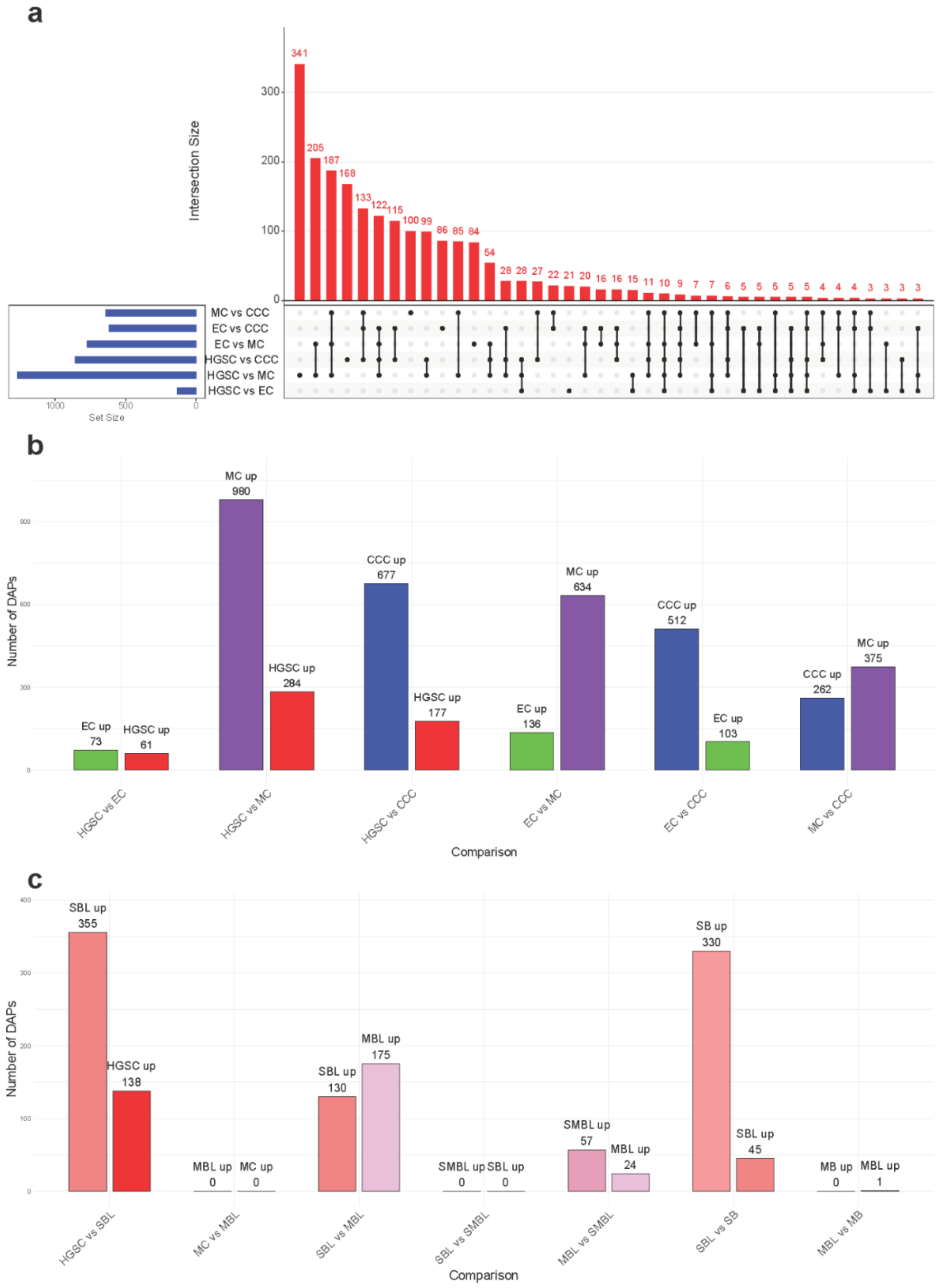

Figure S1

Summary of the differential abundance analysis for pairwise histotype comparisons of malignancies, borderline, benign tumors.

a Upset plot displaying the size distribution of pairwise comparisons along with existing overlaps. **b** Histogram for directionality of abundance for the DAPs for each histotype comparison. **c** Histogram of abundance directionality for HGSC-MC and all BL and BL to B comparisons. B Benign, BL Borderline, CCC Clear-cell ovarian carcinoma, DAP Differentially abundant protein, EC Endometrioid ovarian carcinoma HGSC High-grade serous ovarian carcinoma, MBL Mucinous borderline tumor, MC Mucinous ovarian carcinoma, SBL Serous borderline tumor, SMBL Sero-mucinous borderline tumor.

Supplemental Tables

Table S1

Cohort design and clinicopathologic data after histotype reclassification.

B, Benign; BL, Borderline; CCC, Clear cell ovarian carcinoma; EC, Endometrioid ovarian carcinoma; HGSC, High-grade serous ovarian carcinoma; LGSC, Low-grade serous ovarian carcinoma; MC, Mucinous ovarian carcinoma; NA, Not applicable.

	**HGSC**	**EC**	**MC**	**CCC**	**LGSC**	**BL**	**B**
**Number of samples**	122	42	35	45	8	31	17
**Borderline/benign type**							
Serous						16	11
Mucinous						10	6
Seromucinous						4	0
Endometrioid						1	0
**Patient age**							
Mean	59	61	59	61	54	44	49
Range	34-86	25-86	30-86	40-84	39-77	17-62	23-76
**Overall survival**							
0-2 years	24	9	19	20			
2-5 years	40	13	6	6			
5-10 years	36	8	6	8			
>10 years	22	12	4	11			
**Cause of death**							
Ovarian carcinoma	77	18	19	21			
Other	23	11	13	18			
Alive	22	13	3	6			
**Stage**							
I	26	14	8	12			
II	21	7	2	2			
III	67	16	20	25			
IV	8	5	5	6			
**Dualistic model**							
Type I	0	42	35	45			
Type II	122	0	0	0			
**CA-125**							
<=35	10	2	4	6			
>35	26	4	5	4			
Not answered	48	23	12	9			
Not performed	15	7	6	7			
NA	23	6	8	19			
**Residual tumor size at cytoreductive surgery**							
Tumor free	62	25	14	22			
<=1 cm	21	3	2	2			
>1 cm	29	9	11	8			
Peritoneal carcinosis	8	3	2	1			
NA	2	2	6	12			

Table S2

All unique identified protein accession Ids for searching raw mass spectrometry data with directDIA in Spectronaut along with metadata for all samples.

Table S3
Results from differential abundance analysis after filtering for proteins significantly upregulated (FDR < 0.05, FC > 1.5) and downregulated (FDR < 0.05, FC < -1.5) for all histotypes.

Table S4
Results from differential abundance analysis after filtering for proteins significantly upregulated (FDR < 0.05, FC > 1.5) and downregulated (FDR < 0.05, FC < -1.5) for all pairwise comparisons.

Table S5

Combinations of DAPs yielding the highest AUC for the classification of a histotype/comparison based on SVM models.

Support vecor machine (SVM) models were trained on 80% of the abundance data, and performance tested on the remainingf 20%. SVM models were tested for proteins that had been pre-selected in least absolute shrinkage and selection operator (LASSO) regression, choosing proteins with non-zero coefficients based on lamda min. AUC, Area under the curve; CCC, Clear cell ovarian carcinoma; EC, Endometrioid ovarian carcinoma; HGSC, High-grade serous ovarian carcinoma; MB, Mucinous benign; MBL, Mucinous borderline; MC, Mucinous ovarian carcinoma; SB, Serous benign; SBL, Serous borderline; SMBL, Sero-mucinous borderline.

Panel for histotype/comparison	Protein panel	Protein description panel	AUC
HGSC	GPRC5A, RAB6B, S100A1, CDH6, SSBP1	Retinoic acid-induced protein 3, Ras-related protein Rab-6B, Protein S100-A1, Cadherin-6, Single-stranded DNA-binding protein. mitochondrial	0.854166666666667
EC	ASS1, MAP2K6, PLA2G4A, STRA6	Argininosuccinate synthase, Dual specificity mitogen-activated protein kinase kinase 6, Cytosolic phospholipase A2, Receptor for retinol uptake STRA6	0.804878048780488
MC	KALRN, PPDPF, CALB2, FAM3D, HSPA12A	Kalirin, Pancreatic progenitor cell differentiation and proliferation factor, Calretinin, Protein FAM3D, Heat shock 70 kDa protein 12A	0.840625
CCC	GLRX, PCSK6, ALDH3A2, PLS3, RIMKLB	Glutaredoxin-1, Proprotein convertase subtilisin/kexin type 6, Aldehyde dehydrogenase family 3 member A2, Plastin-3, Beta-citrylglutamate synthase B	0.93287037037037
HGSC vs EC	PDZD8, PTS, VWA2	PDZ domain-containing protein 8, 6-pyruvoyl tetrahydrobiopterin synthase, von Willebrand factor A domain-containing protein 2	0.674285714285714
HGSC vs MC	AGR2, S100A1, PTGS1, KCTD1, BBOX1	Anterior gradient protein 2 homolog, Protein S100-A1, Prostaglandin G/H synthase 1, BTB/POZ domain-containing protein KCTD1, Gamma-butyrobetaine dioxygenase	0.823076923076923
HGSC vs CCC	MET, FAM83B, SPON1, S100A1, CDKN2A	Hepatocyte growth factor receptor, Protein FAM83B, Spondin-1, Protein S100-A1,	0.985714285714286

		Cyclin-dependent kinase inhibitor 2A	
EC vs MC	LYPLAL1, H1-3, CLDN3, CDCA8, KCTD1	Lysophospholipase-like protein 1, Histone H1.3, Claudin-3, Borealin, BTB/POZ domain-containing protein KCTD1	0.767857142857143
EC vs CCC	AP2A2, WARS1, SPATS2, ALDH3A2, CTH	AP-2 complex subunit alpha-2, Tryptophan--tRNA ligase. cytoplasmic, Spermatogenesis-associated serine-rich protein 2, Aldehyde dehydrogenase family 3 member A2, Cystathionine gamma-lyase	0.875
MC vs CCC	AGR3, ST6GALNAC1, CEACAM5, FCGBP, AKR7A3	Anterior gradient protein 3, Alpha-N-acetylgalactosaminide alpha-2.6-sialyltransferase 1, Carcinoembryonic antigen-related cell adhesion molecule 5, IgGFc-binding protein, Aflatoxin B1 aldehyde reductase member 3	0.968253968253968
HGSC vs SBL	SDHAF2, PTBP2, PLBD1, STMN1, PLIN2	Succinate dehydrogenase assembly factor 2. mitochondrial, Polypyrimidine tract-binding protein 2, Phospholipase B-like 1, Stathmin, Perilipin-2	0.945652173913043
SBL vs MBL	S100A1, KLK6, THSD4, CLIC5, KRT17	Protein S100-A1, Kallikrein-6, Thrombospondin type-1 domain-containing protein 4, Chloride intracellular channel protein 5, Keratin. type I cytoskeletal 17	1
MBL vs SMBL	FCGBP, OGN, MAOA, CMBL, PARPBP	IgGFc-binding protein, Mimecan, Amine oxidase [flavin-containing] A, Carboxymethylenebutenolidase homolog, PCNA-interacting partner	1
SBL vs SB	MFSD6, MAPK13, SLC22A18, OSBPL3, TCIRG1	Major facilitator superfamily domain-containing protein 6, Mitogen-activated protein kinase 13, Solute carrier family 22 member 18, Oxysterol-binding protein-related protein 3, V-type proton ATPase 116 kDa subunit a 3	1

Table S6
Significantly enriched (FDR < 0.20) biological processes for the full proteome and for cancer hallmark pathways in gene set enrichment analysis.

Table S7
Significantly enriched (FDR < 0.05) biological processes based on DAPs for respective histotype in a GOEA and number of ocurrences of DAPs.

Table S8

Metadata for survival analysis.

Table S9
All proteins significantly ($p < 0.05$) associated with either high risk ($HR > 1$) or low risk ($HR < 1$) of death form univariate cox regression.

Table S10
Significant proteins from univariate cox regression ($p < 0.05$) that had significant log-rank p-value ($p <0.05$) in both OS and DSS.

Table S11
Significant ($FDR < 0.05$) proteins for multivariate cox regression for log-rank significant ($p < 0.05$) adjusted for LASSO-selected covariates as well as bootstrap validation of survival models.
